# Overview of BPH: Symptom Relief with Dietary Polyphenols, Vitamins and Phytochemicals by Nutraceutical Supplements with Implications to the Prostate Microbiome

**DOI:** 10.3390/ijms24065486

**Published:** 2023-03-13

**Authors:** Kendal L. Stewart, Edwin D. Lephart

**Affiliations:** 1 Neurosensory Centers of America, Inc., Austin, TX 78738, USA; 2 Department of Cell Biology, Physiology and The Neuroscience Center, College of Life Sciences, Brigham Young University, Provo, UT 84602, USA

**Keywords:** prostate, aging, factors, inflammation, polyphenols, phytochemicals, vitamins, nutraceuticals, supplementation, microbiome

## Abstract

Benign prostatic hyperplasia (BPH) is an age-related disorder, which is one of the most prevalent and costly benign neoplasms in men with over 94 million cases worldwide. Starting before or around 50 years of age, there is a linear increase in prostate volume and BPH symptoms, which are influenced by changes in hormonal, inflammatory, growth factors, cell receptor signaling, diet, physical activity, and the microbiome of the prostate that leads to cellular proliferation. While current pharmaceutical or surgical treatments are currently available, each treatment has serious side effects. This dilemma has motived men to seek treatment without negative side effects from medicinal plants such as botanicals, phytochemicals, and vitamins that have established safety records. This narrative overview focuses on several botanicals, phytochemicals and vitamins that are widely used in the treatment of BPH and emphasizes how, in some cases, combinations of these natural ingredients may provide better BPH symptom relief compared to utilization of a single medicinal plant product (monotherapy). Finally, this overview highlights in vitro, in vivo animal studies and mainly clinical data of journal reports published in the past 5 years from January 2018 to January 2023 on BPH and nutraceuticals. Notably, there is an evolving perspective or rethinking of the role that medicinal phytochemicals and natural vitamins usage play; that is, they may hold promise or are likely to alleviate BPH symptoms.

## 1. Introduction

The purpose of this narrative overview is not to provide a comprehensive review, but to update perspectives on BPH which are (1) influenced by changes in hormonal, inflammatory, growth factors, cell receptor signaling, epigenetics, diet, physical activity, and the microbiome of the prostate that leads to cellular proliferation, (2) focused on medicinal botanicals, phytochemicals and certain dietary vitamins that are currently used for BPH treatment, and (3) demonstrated, in some cases, combination therapies of botanical/natural ingredients that may provide better (non-surgical) BPH symptom relief without side effects. This presentation used foundational figures, tables and graphics that depict recent literature results covering (mainly) the last 5 years (from January 2018 through January 2023), where applicable. These topics were explored using the keywords: BPH and hormones, inflammation, growth factors, cell receptor signaling, genetics, diet, physical activity, and briefly the prostate microbiome along with the keywords: equol, beta sitosterol, pumpkin seed extract, saw palmetto, lycopene, stinging nettle, green tea, and vitamins A, C, D, and E. The following databases were utilized: PubMed, Science Direct and Scopus plus Google Scholar. Some references were included without a year interval-range limit, which provided data/background information on various topics. This article is based on previously conducted studies and does not contain new data/results (studies) of human participants or animals performed by the authors.

## 2. Benign Prostatic Hyperplasia (BPH)

**Figure 1:**
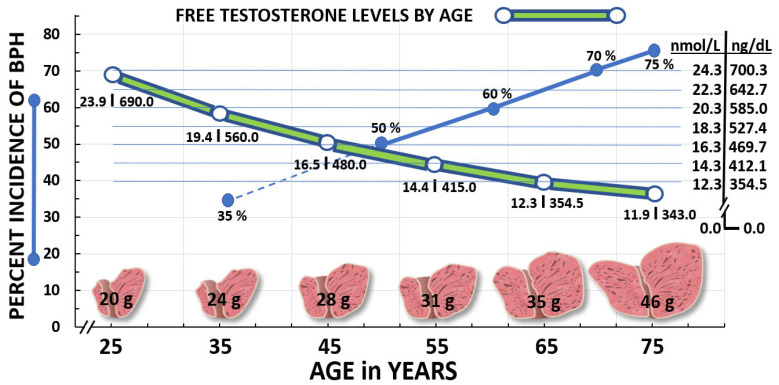
Benign prostatic hyperplasia (BPH) with aging: percent incidence of BPH (left *y*-axis), testosterone levels in nmol/L or ng/dL (right *y*-axis), age in years (*x*-axis). A cartoon at the bottom of the figure displays potential prostate enlargement with gram (g) increases with aging. Free testosterone levels are shown. By middle-age (>35 years old), approximately 30–40% of men have BPH symptoms; see dotted blue line.

Benign prostatic hyperplasia (BPH) is the pathological progression of epithelial and stromal proliferation in the prostate gland with aging and is one of the most prevalent and costly benign neoplasms in men [1,2,3] (Figure 1), with over 94 million cases worldwide in 2019, compared to 51.1 million cases in 2000 [3]. Usually, BPH symptoms begin among middle-aged men (>35 years of age), and by age 50 approximately 50% of men are diagnosed with BPH; thereafter, there is a linear increase in BPH cases with age [1,2,3] (Figure 1). In 2013, Medicare costs in the United States (US) were more than 1.5 billion in US dollars (USD) for BPH-related office and outpatient services [2]. BPH leads to lower urinary tract symptoms (LUTS), which include voiding symptoms (hesitancy, intermittency, poor stream, straining and terminal dribble) and storage problems (nocturia, urgency, and increased frequency) that negatively impact the quality of life (QOL) of patients, family members, and disrupts work performance [1,2,3,4]. Also associated with aging, declining levels of testosterone have been reported in men (associated with higher rates of morbidity) [5,6], sometimes called andropause [7] (Figure 1). However, the cause(s) of decreasing testosterone levels with age have not been clearly identified, and there is uncertainty as to what constitutes optimal testosterone levels for men by age [6,8]. Moreover, investigators reported a decline in serum testosterone levels among adolescent and young adults in the US over the past two decades, especially in individuals with a higher body mass index [9]. The link between androgen and BPH along with other factors will be covered in the next section.

## 3. Factors Influencing BPH

The prostate, like other sex-accessory tissues, is stimulated in its growth and secretory function by the presence of hormones, growth factors via specific receptors, and other influences, which present dynamic elements to the pathophysiology of BPH [2,10,11,12].

### 3.1. Androgens and BPH

**Figure 2:**
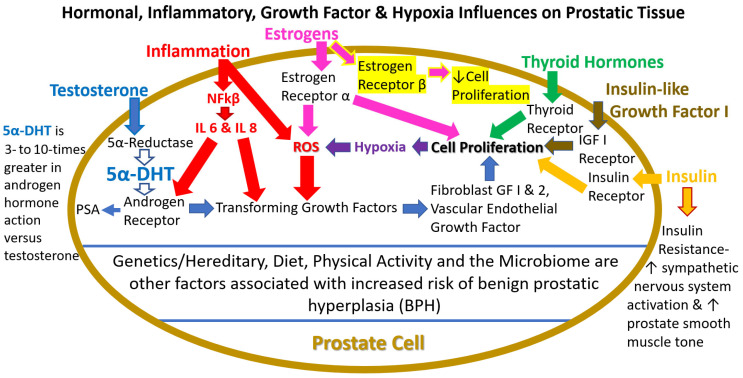
Hormonal, Inflammatory, Growth Factors and Hypoxia Influences on Prostate Cellular Proliferation Observed in BPH. Nuclear Factor-kappa B (NFkβ), Interleukin (IL), reactive oxygen species (ROS), Fibroblast Growth Factors 1 and 2 (FGF 1 and 2).

It is clearly established that androgens play a role in the progression of BPH [2,10,11,12]. Testosterone (a C19 steroid compound) is the primary plasma androgen in men; it appears to function (in this example) as a prohormone, where it is irreversibly converted by the 5α-reductase enzymes to the more potent androgen 5α-dihydrotestosterone (5α-DHT) by the reduction of the double bond at the carbon 4–5 position of testosterone [2,10,11,12] (Figure 2). The 5α-reductase isozymes (type I and type 2) and nuclear androgen receptors (AR) are expressed in epithelia and adjacent stroma cells [10,11,12]. Notably, testosterone’s binding affinity for ARs is approximately 11 nM, while 5α-DHT’s binding affinity for ARs is approximately 1 to 3.5 nM [13]. This suggests that 5α-DHT’s androgen hormone action is approximately 3 to 5 times greater than testosterone and explains, in part, the effects of this potent androgen in BPH [12,13]. In fact, some reports suggest that 5α-DHT’s androgen hormone action is approximately 10 times greater than testosterone where its concentration within the prostate is pronounced [12]. Finally, AR activation via 5α-DHT leads to the increase in growth factors responsible for proliferation. For example, in BPH fibroblasts expressing AR, FGF-2 and FGF-7 are overexpressed [12] (Figure 2).

Conversely, there is clear evidence that the 5α-reductase enzymes increase their expression within the prostate with age and disease states [13]. This suggests that even though testosterone levels decline with age, the conversion to 5α-DHT increases to influence BPH activation and maintenance of cellular proliferation via the AR [10,13] (Figure 2) and downstream growth factors causing inflammation [10,12]. This concept of decreasing testosterone levels, but increasing intra-prostatic expression of the 5α-reductase enzymes with aging and the resulting enlargement of the prostate gland is shown in Figure 1. Thus, the use of 5α-reductase enzyme(s) inhibitors to treat BPH has long been established; however, the negative side effects of these treatments (e.g., impotence, ejaculatory disorders, gynecomastia, depression, anxiety, and the increased risk of more serious prostate cancer) have been reported [2,14,15,16]. Additionally, alpha-1 blockers, commonly used to treat BPH, do not decrease cell proliferation, but work by relaxing prostate smooth muscles to increase urine flow/weak stream; they also have negative side effects (dizziness, lightheadedness, drowsiness, runny/stuff nose and ejaculatory disorders, confusion, and depression) [17]. The side effects and complications of some current pharmaceutical treatments for BPH are shown in Table 1. Finally, the action of androgens binding to the AR, prostate-specific antigen (PSA) levels, as well as changes in the 5α-reductase enzymes with aging/BPH and prostate cancer have been reviewed elsewhere [2,10,11,12,16,18,19,20,21].

### 3.2. Inflammation and BPH

Aging is accompanied by the progressive loss of anatomical structure and physiological function of the body (organs, tissues, and cells) leading to age-related diseases [22,23]. Investigators have proposed that the underpinnings of aging and age-related diseases are based upon low-grade, unresolved, molecular inflammation or oxidative stress events [22,23]. Accumulated data strongly suggests that continuous (chronic) upregulation of pro-inflammatory mediators leads to inflammation [12,13,22,23,24,25]. This is regulated by different types of cytokines such as interleukin-1 (IL-1, IL-5, IL-6), interferon-gamma (IFNγ), tumor necrosis factor alpha (TNF-α), chemokines (such as IL-8), vascular cell adhesion molecule 1 (VCAM-1), monocyte chemoattractant protein (MCP-1), prostaglandins, enzymes (like matrix metalloproteinases (MMPs), cyclooxygenase 2 (COX-2), growth factors (GFs), and inducible nitric oxide synthase (iNOS), etc. [12,13,22,23,24,25]. These are activated during the aging process due to an age-related redox imbalance where many pro-inflammatory signaling pathways [including MAPK and nuclear factor-kappa B (NFkβ)] lead to the formation of reactive oxygen species (ROS) [12,13,22,23,24,25].

The chronic inflammatory process: the link between the initial cause and the growth factor stimulated hyperplasia of glandular cellular proliferation seen in BPH is clear [12]. However, the initial stimulus for the inflammatory process is unknown; however, several potential factors may include bacterial or viral infections, hormone changes, dietary factors, autoimmune responses, urinary reflux into the prostate collecting ducts, and the potentially the prostate microbiome [10,12,26,27]. While there is some evidence that AR expression is elevated in certain cellular compartments, AR signaling within stromal cells alters intercellular signaling and changes in epithelial AR expression leads to paracrine signaling and/or chronic inflammation that further aids in stromal or epithelial proliferation in BPH [20]. Presumably, the initial stimulus involves activated T cells from stromal cells that release proinflammatory cytokines, chemokines, and interleukins (ILs) that are responsible for cellular damage [10,12]. Specifically, IL-8 is thought to be key in the induction of fibroblast growth factor-2 (FGF-2) expression leading to cellular proliferation [10,12]. This mechanism is a self-perpetuating cycle, where chronic inflammation leads to a progressive increase in prostate volume [11,12]. Furthermore, it has been shown that IL-8 plays a role in prostatic cancer progression via promotion of androgen receptor-independent mitogenic pathways [28]. Finally, prostate cellular proliferation causes a localized hypoxic environment that stimulates the release of reactive oxygen species (ROS) [10,11,12,13]. In turn, the interleukins and ROS promote (a) the release of various growth factors [fibroblast growth factor-1 (FGF-2), fibroblast growth factor-7 (FGF-7) and transforming growth factor—beta 1 (TGF-β1)], and (b) the generation of a new vascular supply (via vascular endothelial growth factors) [10,11,12]. This ‘malicious cycle’ (Figure 2) demonstrates the presence and proliferation of inflammation, whereas use of 5RIs or alpha-1A blockers may not lead to BPH symptom reduction [12,14] compared to other natural active ingredient(s) such as saw palmetto that reduce 5α-reductase enzyme activity, etc., which does not have side effects [17].

### 3.3. Estrogens and BPH

Estrogens have been known and studied for a century. These compounds are named according to the number of hydroxyl side-groups in the molecule [29]. All natural estrogens arise by the enzymatic removal of a carbon atom from androgen precursor molecules (such as testosterone), a process termed aromatization, which is a product of the CYP19A1 gene present in prostatic stromal cells [10,11,12,29,30]. Estrogens transmit their chemical messages to nuclear estrogen receptors, estrogen receptor α (ERα) within prostate stromal tissue and ERβ is expressed in basal epithelial cells [10,11,12].

While 5α-DHT has potent actions within the prostate (and other tissue sites), 17β-estradiol is the most potent sex steroid hormone in the body [29,30] and estrogens, in general, are, mole for mole, 100 to 1000 times more biologically active or potent compared with their parent androgens (e.g., testosterone) [30]. 17β-Estradiol has very high and almost equal affinity for ER-α and ER-β via competition binding studies, where the Kd is 0.13 nM and 0.15 nM, respectively [13]. Thus, presumably, estradiol within the prostate binds equally well to both ER-α and ER-β.

In comparison to the above, the production of prostatic 5α-DHT increases with aging due to the increased expression of the intraglandular 5α-reductase enzymes; there is also evidence that the aromatase enzyme that converts androgens to estrogens significantly increases within adipose tissue with aging in men. From early investigations by Hemsell et al. in 1974, both women and men demonstrated that with advancing age, there is a progressive and significant increase in the efficiency (by 2- to 4-fold) with which androgens are converted to estrogens, suggesting that increased expression of the aromatase enzyme was responsible for these findings [31]. Later, molecular biology studies by Bulun and Simpson in 1994 confirmed this notion and extended these findings that aromatase gene expression was greatest in the buttocks and thighs followed by the abdomen with advancing age [32]. Correlations between visceral adipose tissue, increased inflammation, and estrogen levels in aging or obese men have been reported [10,26,27]. Finally, in a clinical study, men with obesity and type 2 diabetes displayed increased prostate volumes [33], suggesting a link between enhanced adipose deposition, aromatase enzyme activity, high insulin levels and BPH.

However, in 2009 Ellem and Risbridger reviewed the dual, but opposing role of estrogens in the prostate [34]. In brief, they reported (1) that estrogens are essential for normal tissue homeostasis within the prostate, (2) the importance and differential roles of prostatic ER subtypes and most significantly, (3) that activation of ER-α leads to aberrant proliferation, inflammation, and the development of premalignant lesions, while in contrast, the activation of ER-β is critical in prostatic stromal–epithelial cell signaling and mediates anti-proliferative effects that balance the proliferative action of androgens on epithelial cells [34]. This hypothesis of critical endocrine and paracrine estrogen signaling in the prostate has been updated and reviewed elsewhere [35], where ERβ-agonists may provide treatment for BPH [35].

**Figure 3:**
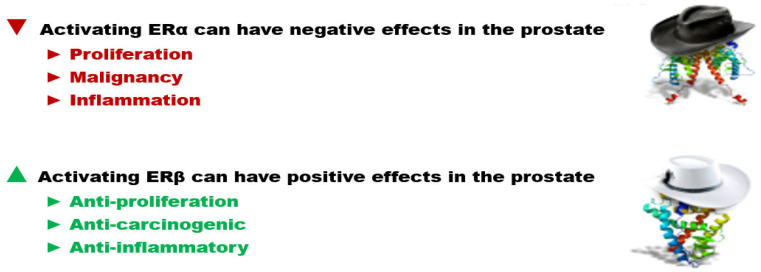
Estrogen receptor alpha (ERα) and estrogen receptor beta (ERβ) actions in the prostate. Using a Western cowboy theme, the black hat displays negative effects while the white hat displays positive effects. The G protein-coupled ER (GPER) also has been implicated in benign prostatic hyperplasia (BPH), but much less data are known about this link compared to the ERα and ERβ actions in the prostate. Adapted [13] with permission http://dx.doi.org/10.4236/ojemd.2014.41001, accessed on 13 February 2023.

Finally, there is evidence that the stimulation of inflammation in BPH by estrogens can be mediated by the membrane ER G protein-coupled receptor 30 (GPR30) or G protein-coupled ER (GPER), which are also expressed in prostate stromal cells [35]. Therefore, these data emphasize the importance and complexity of estrogen hormone action within the prostate and highlight the known capacity of estrogens to exert both beneficial and adverse effects via ER-β compared to GPER and ER-α, respectively [34,35] (Figure 2 and Figure 3).

### 3.4. Thyroid Hormones and BPH

Thyroid hormones (THs) are involved in cellular growth, metabolism, and differentiation [35,36]. Their effects are mainly mediated by binding nuclear TH receptors (TRs) that are highly expressed in the prostate [36] that activates TH response elements (TREs) in the promoter of TH target genes [35,36]. However, non-classical or nongenomic effects of TH have also been described [35].

Aging is associated with changes in thyroid function, where hyperthyroidism is less common than hypothyroidism [35]. However, several studies have examined the role of THs in the development of BPH and prostate cancer [35,36,37,38,39]. For example, some clinical studies found higher cases of BPH with increased plasma T3 levels compared to euthyroid men [35]. Eldhose et al., 2016, found significantly increased levels of free T3 (FT3) and free thyroxine (FT4) and decreased levels of TSH in patients with BPH compared with controls [37]. Additionally, a recent meta-analysis study showed that hyperthyroidism was associated with higher risks of prostate cancer [36]. Other studies also indicated that T3 has an important role in the regulation of growth and differentiation of LNCaP cells and PSA expression [35]. Moreover, Lee et al., 2019, examined the relationship between thyroid hormone levels and BPH and found that total prostate volume and international prostate symptoms score (IPSS) values were significantly related to FT3 levels [39].

From one perspective, there is a clear relationship between thyroid hormones and BPH, since a recent study by Miro et al., 2022, showed that thyroid hormones and androgen signals mutually interplay and enhance inflammation and tumor formation in prostate cancer [40]. Conversely, some investigators report an unlikely link between thyroid hormones and BPH [41,42], especially since a large clinical study showed that hyperthyroidism is not a significant risk for BPH, when adjustment for comorbidities and covariates were performed [43]. In conclusion, although a relationship between TH and prostate seems apparent, the mechanisms by which this occurs warrant further investigations to determine the actual link between thyroid hormones and BPH/prostate cancer.

### 3.5. Insulin-like Growth Factor I, Insulin and BPH

Insulin-like growth factor 1 (IGF-1) is produced mainly in the liver by growth hormone (GH) signaling and is necessary for normal physical growth, especially for bone and muscle [12,35]. However, IGF-1 levels decline with aging, whereas increased levels are associated with several types of cancer and type 2 diabetes [35,43]. Early studies showed an association between IGF-1 and BPH risk [44,45]. Since then, additional evidence has demonstrated that the GH-IGF-1 endocrine axis plays an important role in the growth and development of the prostate [35]. For example, the binding of IGF-1 to the IGF-1 receptor (IGF1R) or the insulin receptor (IR) in the prostate gland activates the phosphoinositide 3-kinase (PI3K)/protein kinase B (AKT) pathway and RAF/MAPK pathway, which promote cell survival and proliferation [35] (Figure 2). This concept is supported where Sreenivasulu et al. (2018) showed that when gene expression of IR and IGF-1 increased, prostate size also increased [46]. More recently, Matsushita et al., 2022, demonstrated the link between gut IGF-1 and the prostate axis, where IGF-1 was shown to be directly involved in prostate carcinogenesis [47]. In this case, with the connection to gut IGF-1, the authors suggested that optimal diets and/or nutritional supplementation may ameliorate prostate disorders including BPH and prostate cancer [47]. Finally, in a matched-controlled clinical study, Ali et al. in 2014 showed that IGF-1 played an important role in developing BPH in type 2 diabetic patients with low PSA levels (between 0.7 and 2.4 ng/mg) [48].

Insulin is a peptide hormone produced in beta cells of the pancreas islets and is considered the main anabolic hormone of the body for glucose transport into cells controlling blood glucose levels [12,35]. A role for insulin as a risk factor has been proposed for BPH, and several studies have examined components of metabolic syndrome (MetabS) in the pathogenesis of BPH and LUTS, which have been reviewed elsewhere [12,33,35]. Notably, MetabS covers five factors, in general: (i) obesity, (ii) increased blood glucose, (iii) decreased high-density lipoproteins (HDL) cholesterol, (iv) increased triglycerides and (v) hypertension [35]. Insulin is thought to act via the ILGF-1, whose receptor is highly expressed in prostate stromal cells [49] (Figure 2). For example, hyperinsulinemia has been shown to enhance prostatic epithelial cell proliferation in vitro, and conversely, hypo-insulinemia decreases prostate volume [34]. Epidemiological studies have shown an increased incidence of BPH in patients with diabetes [33,35]. In line with these findings, in general, studies have shown that patients with serum insulin levels >13 mU/L have a greater prostate volume and annual BPH growth rates compared with those with insulin levels at or below 7 mU/L [35,50,51]. Moreover, a recent retrospective study carried out in 900 patients reported that, after correction for age, insulin levels and insulin resistance were significantly associated with prostate volume, that predicted BPH/LUTS clinical progression [52]. Additionally, diet-induced hyper-insulinemia and essential hypertension have been reported as key factors in BPH patients [53]. Thus, the relationship between IGF-1, insulin and BPH appears certain based upon the various journal reports and clinical investigations [12,35,43,44,45,46,47,48,49,50,51,52,53], which points to potential ways to therapeutically confront prostate disorders.

### 3.6. Epigenetic Factors: Diet/Nutrition, Physical Activity and BPH

**Figure 4:**
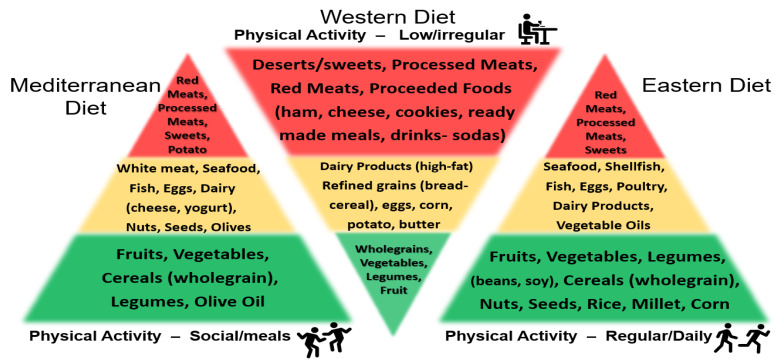
Comparison Among Mediterranean, Western and Eastern Diets. **Mediterranean Diet**: High intake of olive oil (principal source of fat), vegetables (leafy green vegetables, onions, garlic, tomatoes, and peppers), fresh fruits, whole grain cereals, nuts, and legumes. Moderate intake of fish, and other seafood, poultry, eggs, dairy products (mainly cheese and yogurt), low intake of red wine and very low intake of red meat, potatoes, processed meat, red meat, refined carbohydrates, and sweets. **Western Diet**: High take of pre-packed highly processed foods, red meat, processed meat, high-sugar drinks, candy, sweets, fried foods, conventional-raised animal products (chickens, pigs, turkeys), butter, high-fat dairy products, refined grains, eggs, potatoes, corn (high-fructose corn syrup), and low intake of fruits, vegetables, whole grains, fish, nuts, and seeds. **Eastern Diet**: High intake of plant-based foods (source of protein from vegetables- bean sprouts, spinach, eggplant, bok choy, cabbage, kale, snow peas, leeks, and mushrooms). Fruits and legumes- grapes, melons, cherries, dates, mangoes, etc.; steamed or stir-fried produce along with nuts, seeds, beans (soy, mung), lentils, tofu, or tempeh, plus, rice and whole grains. Moderate intake of fish (dependent upon country’s coastline), dairy, eggs, and poultry. Very low intake of meat, processed meat, refined carbohydrates and sweets.

While conventional drugs such as alpha-1 blockers and 5α-reductase inhibitors (5RIs) have been used to treatment BPH, the adverse side effects associated with their usage have led men to search for alternative natural plant-derived remedies to manage LUTS/BPH disorders. Additionally, although surgery is an option, the increased cost and risk associated with it are not chosen by (especially younger) men as a viable course of treatment [54,55]. In 2011, Alegria-Torres et al. published a landmark review on epigenetics and lifestyle, where they outlined environment and lifestyle factors such as diet/nutrition, behavior (physical activity, sleep, working habits, stress, etc.), and smoking (vaping), and alcohol consumption that might affect human health [56]. In this regard, more than 10 years ago, early studies suggested that the lack of exercise and a poor diet were associated with LUTS/BPH [57,58]. More recently, in 2022 La Vignera and Basile presented a report entitled “Diet and prostate health: an underrated tool?”, which reviewed how dietary changes can notably impact prostate health and improve the benefit of traditional medical care [59]. While diet has become a focus to enhance human health, the attention on what types of diets yield the best outcomes is paramount from the perspective of consumers. The “Mediterranean or Eastern” diets versus a “Western diet” have gained popularity and promise not only for the management of LUTS/BPH, but to increase the general health status and well-being to address many other diseases and disorders [60,61,62,63,64]. For example, the Mediterranean diet is one of the most widely described and evaluated dietary patterns in scientific literature [61]. It is characterized by high intakes of vegetables, legumes, fruits, nuts, whole grains, fish, some olive oil and a moderate intake of red wine, where most proteins and fats are derived from vegetable sources [61]. Additionally, the Eastern diet includes foods that contain β-carotene, vitamin C and, vitamin E found in vegetables, fish/seafood, lycopene found in tomatoes, and polyphenols/isoflavones found in legumes (beans, soy, etc.) [64]. Conversely, the Western diet contains refined carbohydrates, red meat, processed meats, fats/lipids/cholesterol, prevalent in high-income countries, which increase sympathetic nervous system, oxidative stress, and inflammation and low intake of fruits and vegetables [60,61,62,63,64,65] (Figure 4). Additionally, shown in Figure 4 are the relative levels of physical activity associated among the three diets.

Notably, the relationship between dietary patterns and risk factors for LUTS/BPH, (but also for erectile dysfunction and prostate cancer) have been reported and reviewed elsewhere [66,67,68,69,70,71,72]. From these various reviews, the risk factors for BPH include (a) age [60,65,66,69], (b) smoking (vaping) [69], (c) obesity/overweight [69,72], (d) ↑ insulin [34,52,53,57,61,69], (e) ↑ lipids [61,69,72], (f) diabetes [33,35,69], (g) hypertension [53,69], and (h) depression [69]. Conversely, vitamins A, C, D and E are associated with decreased BPH symptoms [59,64,67,69,70,71,72]. Among the vitamins, D appeared to have the greatest impact on BPH followed by vitamins C, E, and then, A [64,69,72]. This was especially the case with vitamin D deficiency, where prostatic inflammation was associated with the activation of the NFkβ, IL-6, and STAT3 pro-inflammatory pathways [70].

To determine whether a high-fat diet induced BPH inflammatory responses via the STAT3/NFkβ and Nrf2-mediated oxidative stress pathways, Li et al. in 2019 reported alterations in inflammatory, apoptosis, and oxidative stress parameters using a rat model [73]. Specifically, compared to controls, the prostate levels of COX-2, iNOS, TNF-α, IL-6, malodialdehyde (MDA), TGF-β1, and monocyte chemotactic protein-1 (MCP-1) were significantly increased, while glutathione peroxidase, glutathione reductase, glutathione, and superoxide dismutase (SOD) were significantly decreased. Additionally, the expression of STAT3 and NFkβ was significantly increased, while levels of Nrf2 were markedly decreased, which demonstrated that a high-fat diet contributed to BPH in this rat model via the STAT3/NFkβ- and Nrf2-mediated oxidative stress pathways [73]. As a point of reference, it is known that a high-fat diet in humans is associated with obesity and dysbiosis of the gut microbiome [74].

Furthermore, in vivo animal studies revealed that ghrelin aggravates BPH in rats and smooth muscle contraction in human prostate tissue [75]. Recall, ghrelin is known as the hunger hormone (produced mainly by the stomach) to increase food intake and to help the body store fat. In this study, Wang et al. in 2019 showed that ghrelin upregulated specific genes (BMP6, TTK, CCNE1 and FOXM1) that induced proliferation resulting in prostate enlargement (via stromal cell growth) and aggravated smooth muscle contraction (via GAL and RTKN2) in human prostate tissues [75]. Another study by Gu et al. in 2021 examined the role of a high-fat diet in the gut microbiota and ghrelin, as well as their contribution to BPH in mice and prostate tissue from BPH patients [76]. The obtained results suggested that alterations from a high-fat diet with changes in the gut microbiota may associate with ghrelin to play an important role in activation of the JAK2/STAT3 pathways to cause inflammation and BPH development in mouse and human prostate tissue [76]. The authors speculated that the gut microbiota and ghrelin might be targeted for future drug development for BPH.

**Figure 5:**
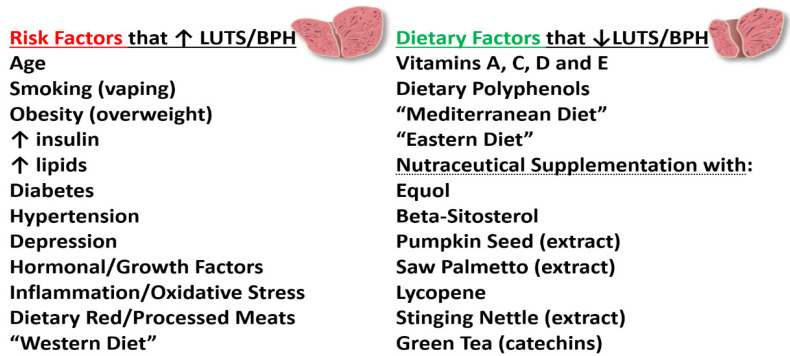
Risk Factors that increase LUTS/BPH and Dietary Factors that decrease LUTS/BPH. LUTS = lower urinary tract symptoms; BPH = benign prostatic hyperplasia. Cartoon of increased prostate volume on the left and decreased prostate volume on the right.

In 2017, Eleazu et al. proposed that management of BPH by dietary polyphenols may be an alternative to existing therapies [77]. In their review, several factors such as inflammatory mediators, hormonal, environment, and oxidative stress (via free radicals) mechanisms are clearly indicated in the development and progression of BPH. In brief, dietary polyphenols have gained increased public interest in recent years due to their anti-inflammatory properties via multiple mechanisms through inhibition of mitogen-activated protein kinases (MAPK) signaling pathways and NFkβ and AP-1 transcription factors, blocking the production of inflammatory cytokines and chemokines, and suppressing the activity of COX-2 and iNOS [77]. Additionally, a more recent report in 2022 by Hu et al. reviewed the properties of polyphenol/flavonoids that can interfere with androgen synthesis and metabolism to regulate androgen hormone actions, thus providing a nutritional intervention to improve androgen disorders such as BPH [78]. In connection with this perspective, soy isoflavones have been studied and reviewed for their protective effects on BPH and prostate cancer [60,61,69,72,79] which include all the positive multiple mechanisms of actions outlined by Eleazu et al. above [77]. Likewise, other reviews on dietary polyphenols/flavonoids for their anti-inflammatory and anti-allergic studies have been reported recently [79,80]. Furthermore, a relatively new isoflavonoid, equol, has been reported to protect against LUTS/BPH, which is gaining increased research and public attention in this regard [13,72,81]. In conclusion, diet/nutritional and physical activity are epigenetic factors that provide alternative remedies/therapies for the management of LUTS/BPH. However, further research is warranted to determine the extent to which prevention and treatment of chronic inflammatory conditions such as BPH can benefit from these interventions. Risk factors that increase LUTS/BPH and dietary factors that decrease LUTS/BPH such as vitamins and polyphenols/phytochemicals that hold promise or are likely to alleviate BPH symptoms are displayed in Figure 5.

### 3.7. Microbiome and BPH

In brief, our understanding of the microbiome has been facilitated by the evolution of various technologies and tools such as DNA content (metagenomics), RNA expression (metatranscriptomics), protein expression (metaproteomics) and small molecule metabolites (metabolomics); over the last two decades, these technologies have revealed the influence of the microbiome on human health and disease [82,83,84]. From the Human Microbiome Project (HMP) sponsored by the National Institutes of Health (NIH), a 2007 extension of the Human Genome Project) highlights how a person’s microbiome is critical for immune system development, function, and homeostasis for health [82,83,84], whereas dysbiosis (altered composition of microbes has a cascading impact on the immune system) can contribute to the susceptibility to infectious disease and chronic illness among human organ systems [83,84]. For example, dysbiosis is associated with increased oxidative stress, inflammation, and damage to DNA repair mechanisms [83,84]. The results from the HMP revealed that there are approximately 2,000,000 microbial genes, approximately 100 times the 20,000 human cellular genes [83,84]. From another perspective, the human body contains trillions of microorganisms outnumbering human cells by 10 to 1. Because of their small size, microorganisms make up only about 1 to 3 percent of the body’s mass (in a 200-pound adult, that is 2 to 6 pounds of bacteria) [83,84].

The most studied microbiome to date is the human gut, and it has been implicated in brain, cardiovascular, respiratory, and skin health [83,84,85]. Additionally, the following conditions or disorders are associated with the gut microbiome: aging, allergic/immune, cancer, diabetes, metabolic syndrome, gastrointestinal, infection, and rheumatoid arthritis [84,85].

Subsequently, the microbiome of other organs/tissue sites has been investigated, including the prostate, where the microbiome-related evidence for BPH/LUTS has been studied in recent years and is beginning to be appreciated only now [22,23]. The urinary tract, once thought to be sterile, has now been shown to harbor bacteria [22,23,86]. The potential role of microorganisms in the urinary tract in the pathogenesis of urogenital disorders, including BPH/LUTS from preliminary studies, suggests how inflammation may be linked to the microbiota [22,23,86].

For example, Frǿlund et al. in 2018 studied healthy men (*n* = 46) and found the main bacterial taxa (*Gardnerella*, *Lactobacillus*, *Sneathia*, *Finegoldia*, *Alphaproteobacteria*, *Prevotella* and *Enterococcus*) in first-voided urine samples using the bacterial 16S ribosomal RNA gene high-throughput next-generation sequencing (NGS) platform to identify microbial profiles for taxonomy [87]. In another clinical study of 30 patients, urine and fecal samples were analyzed by bacterial 16S ribosomal RNA NGS to identify microbial profiles for taxonomy that were correlated with the international prostate symptom score(s) (IPSS) for LUTS [88]. The results showed a correlation between *L. Blautia* (an anaerobic fecal bacteria with probiotic characteristics) and LUTS. Apparently, *L. Blautia* had a protective effect against LUTS in patients with moderate or severe LUTS symptoms [88,89]. In 2020, Bajic et al. analyzed the microbial characteristics using 16S ribosomal RNA NGS in urine of 28 men with LUTS symptoms (undergoing surgery for BPH; group A) and compared this with 21 men with LUTS symptoms not undergoing surgery (group B) and found the presence of dissimilar microbiota in the urethra and bladder regions between the two groups [90]. The results reported by Bajic et al. suggested that urine was adequate to sample the bladder microbiome, and the increased IPSS values were associated with significantly higher odds of detectable microbiota components in group A versus group B patients [90]. Furthermore, clinical reports by Jain et al. in 2020 and Lee et al. in 2021 support this notion, in part, where some bacterial genera correlated with high IPSS values as well as biomarkers of inflammation and cellular damage associated with BPH [91,92]. In fact, the report by Jain et al., 2020 identified *Escherichia coli* (phylum *Proteobacteria*) as a common constituent of benign prostate hyperplasia-associated microbiota that induced inflammatory and cellular DNA damage by prostate tissue staining in a majority of the 36 men studied [91]. Notably, they showed that BPH-associated *E. coli* induced NFkβ signaling (in vitro) and DNA damage in the prostate epithelial cells samples [91]. While in the Lee et al., 2021 study, 77 men with BPH, plus 30 controls subjects were examined [92].

The findings by Jain et al. in 2020 were confirmed, in part, by a Yin et al. study in 2021 that examined 12 men with prostate cancer, 19 men with BPH and 12 healthy men that served as controls, where analysis of urine flora via DNA sequencing of the 16S ribosomal RNA in the V4 region was performed. The results showed that increased *E. coli* levels were accompanied by decreased levels of probiotics such as *Lactobacillus helveticus* and *Lactobacillus iners* in men with prostate cancer and BPH [93]. This suggested an imbalance of the urine flora that may be used as new biomarkers in prostate disorders. However, some other studies suggested that urine samples may not adequately characterize the male bladder microbiome, and thus, the link between prostate microbiota and BPH symptom severity may not be robust [86].

One report examined the gut–prostate axis, where dietary influences such as a high-fat diet caused gut dysbiosis and gut bacterial metabolites such as short-chain fatty acids and phospholipids that enter the systemic circulation in the promotion of prostate cancer growth [94]. Another report by Piwowarski et al. in 2021 attributed the role of the microbiota via the influence of polyphenol’s postbiotic metabolites in providing protective mechanism(s) in the pathophysiology of prostate disorders [95]. Finally, in 2022, Kumar et al. reviewed the protective effects of green tea catechins on prostate cancer chemoprevention, and the role the gut microbiome may play (by the metabolism of catechins by gut microbes) to make them more accessible to the body to exert their beneficial health effects [96]. Thus, examination of the prostate microbiome is an emerging area of study that warrants further investigation, where the microbial environment may improve patient outcomes with greater understanding and knowledge of the inflammatory and protective mechanisms of the microbiota within the bladder and prostate [86,87,88]. Otherwise, the current evidence linking the prostate microbiome to BPH is suggestive (not compelling or clear), and therefore, difficult to utilize in clinical practice [86].

## 4. Nutraceutical Therapies for BPH

According to the Centers for Disease Control and Prevention (CDC), in 2017–2018, almost 60% of adults 20 years old and over reported using herbal dietary supplements (HDS) in the past 30 days in the United States [97]. HDS use increased among all adult age groups in the US, but especially in individuals 60 years old or older reported taking four or more HDSs, and over 80% of women within that age range were the most likely to use HDSs [97]. Moreover, HDSs have become popular as adjunct therapies for COVID-19, where one systematic review of 12 randomized control trials found that consumption of HDSs and zinc sulfate may significantly benefit COVID-19 recovery [98], but additional studies are warranted to confirm this finding. There is even a report that green tea compounds may potentially protect against COVID-19 [99]. In this regard, consumers were drawn to HDS products to enhance their immunity and general health [100]. The rise in sales, in general, suggests increased acceptance and favorable views of HDS by the American public, who were willing to incorporate complementary, alternative, and integrative treatments to address their health needs by natural products from plant sources, vitamins, minerals and nutrigenomic testing.

In 2014, Buckingham estimated that there are more than 200,000 natural products that have been identified [101]. Lui reported that thousands of individual dietary phytochemicals have been identified, for example, in fruits, vegetables, whole grains, legumes, nuts, and other food/plant sources, but a large percentage of them remain unknown [102]. Notably, the potential clinical usefulness of polyphenols and natural compounds for the treatment of BPH has been recently reviewed [103,104,105,106].

While current pharmaceutical or surgical treatments are currently available, each treatment has potential serious side effects [14,15,21]. This dilemma has motived men to seek medicinal plants such as botanicals, phytochemicals, and vitamins (without negative side effects and established safety data) for BPH symptom relief [103,104,105,106]. In fact, recent reports indicated that younger men seek natural therapies over pharmaceutical or surgical treatments for BPH [3,4], which suggests a greater awareness of remedy options including combination therapies that may include both in the management of BPH symptoms [103,105]. Thus, this section provides an overview focused on natural active ingredients that have been utilized as monotherapies or in combinations for the treatment of BPH that include: 4′,7-isoflavandiol (equol), beta sitosterol, pumpkin seed extract, saw palmetto, lycopene, stinging nettle, and green tea. Lastly, it is beyond the scope of this overview to cover all or the most popular natural ingredients in a comprehensive manner, but the focus will be, for the most part, on recent studies within the last 5 years with some prior reports cited that provide background information.

### 4.1. 4′,7-Isoflavandiol (Equol) and BPH

4′,7-Isoflavandiol (Equol) is classified as a polyphenol and isoflavonoid compound, which is relatively new to nutraceutical applications since it was discovered in the early 1908s in humans [107,108,109] (see “equol hypothesis” below). Isoflavonoids are well-known plant secondary metabolites that have gained research attention due to their multiple nutraceutical and pharmaceutical applications [103,105,107]. In plants, isoflavonoids play an important role in the plant’s defense that can provide a competitive advantage to survive and flourish under environmental challenges such as heat and drought [108]. In the late 1990s, the ‘equol hypothesis’ was proposed, which implied health benefits in humans with adequate equol levels (for protection against breast and prostate cancer); thereafter, increased research attention on this isoflavonoid compound has flourished [103,105,107,110,111].

**Figure 6:**
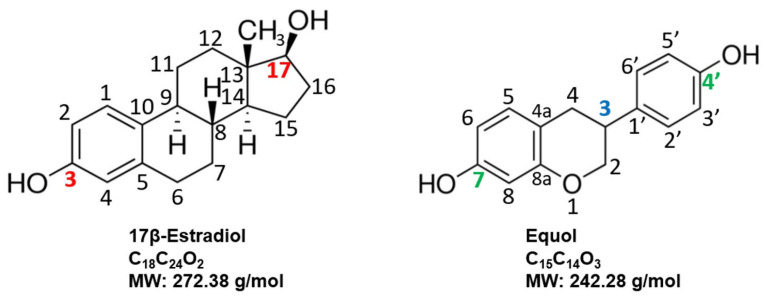
Comparison of the chemical structures, molecular formulas, and molecular weights between 17β-estradiol and equol. Equol (with the chiral carbon at position 3 is shown in blue) represents racemic equol (which is 50% S-equol and 50% R-equol). For both compounds shown, the functional hydroxyl (OH groups), which enables binding to ERs, are indicated at carbon 3 and 17 for 17β-estradiol in red and at carbon 7 and 4′ for equol in green.

Equol has a chiral center at carbon 3, and thus, can exist in two mirror image forms or enantiomers (S-equol and R-equol), which (1) are both biologically active and can specifically bind free 5α-DHT, an important aspect for BPH treatment [13,107,109], (2) can be found in plants, food, and animal products [13,110], (3) are classified as phytoestrogens that act like selective estrogen receptor modulators (SERMS) that bind ERβ receptors in the prostate to protect against BPH [13,110,111,112,113], (4) provide an inhibitory action on the aromatase enzyme that is responsible for estrogen biosynthesis and cellular proliferation [114,115] and, (5) can be biosynthesized to high purity for commercial use in human topical and oral applications [116]. Comparisons of the chemical structures of 17β-Estradiol and Equol are shown in Figure 6.

At this point, it is important to note that the terms soy, isoflavones and phytoestrogens can be used collectively, since soybeans are a high source of isoflavonoids, and in turn, flavonoids are phytoestrogens. Thus, these terms can be used interchangeably [116]. Conversely, from a scientific perspective isoflavones should not be equated with estrogen, and soy foods should not be equated with isoflavones [116,117], which have been shown to be safe [117]. Finally, the controversies and misinformation about phytoestrogens have been addressed in a major recent review in detail [116]. Since everyone is exposed to and consumes phytoestrogen molecules everyday regardless of age, gender, or geographic location around the world, how we understand their effect(s) is a matter of perspective [116].

Earlier studies showed that persons, who could produce equol in response to consuming soy isoflavones, were classified as “equol producers”, which implied health benefits [13,109,112]. It should be noted that this terminology represents individuals that display equol levels of around 10 to 20 ng/mL or more, which is an arbitrary biomarker [13], whereas in comparison to most other mammals (except pigs) the levels of equol range from 800 to over 2500 ng/mL [13]. Miyake et al., 2022, reviewed prior studies that showed that supplementing healthy men (*n* = 28) with dietary soy isoflavones resulted in no changes in estrogen or testosterone levels, but an 18 percent decline in serum 5α-DHT levels was observed versus before supplementation [26], which suggested that in equol producers this may account for lower BPH levels in Asian men [13,110,118,119].

Additionally, the presence of estrogen-related-receptor gamma (ERR-γ) appears to play an important role in prostate health, where it has been shown to slow proliferation/growth in androgen-sensitive prostate cancer cells [13]. In fact, equol has affinity for ERR-γ and is known to increase the transcriptional activity of ERR-γ along with binding to ERβ to increase positive estrogen-like influences in the prostate [13,120].

**Figure 7:**
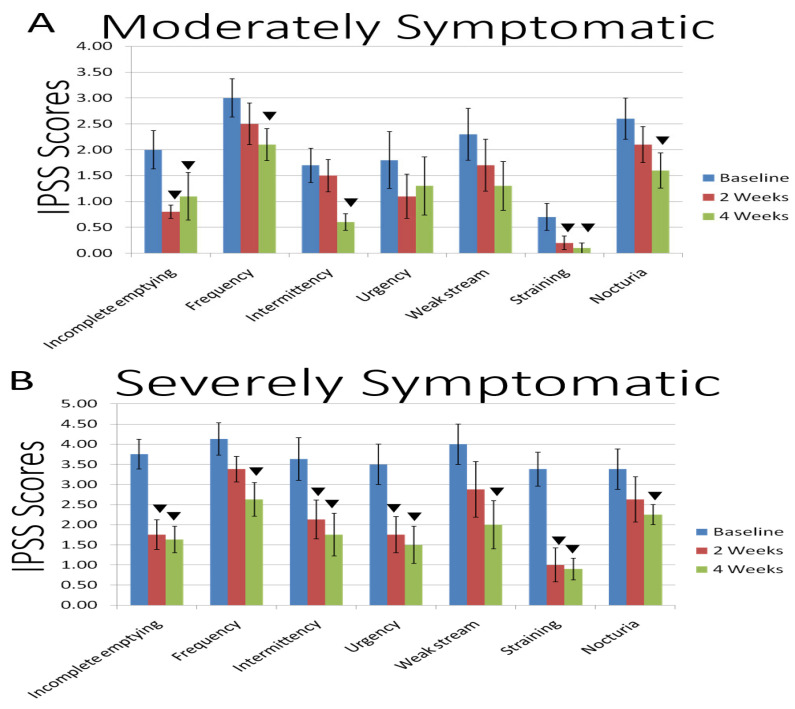
IPSS index of Moderately Symptomatic (**A**), *n* = 10 and Severely Symptomatic (**B**) *n* = 8 mid-aged men across the seven qualified parameters from baseline, at 2 weeks and at 4 weeks of treatment. ▼ = Significantly decreased IPSS values at 2 weeks and/or 4 weeks compared to baseline levels. Adapted [13] with permission http://dx.doi.org/10.4236/ojemd.2014.41001, accessed on 13 February 2023.

In other clinical randomized, double-blind studies (60 and 41 subjects, respectively), examined the safety and pharmacokinetics of equol evaluating dosing from 10 to 320 mg per day in healthy adult volunteers [121]; however, the results of these studies have not been published in the public domain. Nevertheless, previous clinical investigations on equol’s pharmacokinetics indicated that (1) gastrointestinal (GI) absorption is approximately 80% in humans and (2) the half-life (T ½ of either S- or R-equol) after oral ingestion was 6 to 8 h [13,109] where in non-equol producers baseline equol levels ranged between 2 and 4 ng/mL, but increased from 121 to 137 ng/mL after approximately 45 min with a single 6 mg equol dose (*n* =18) [118] (see Figure 7A,B).

The overall IPSS values from a pilot clinical study that evaluated equol as a potential treatment for BPH symptoms are shown in Figure 7A,B and Table 2 (below). Equol at 6 mg was taken by oral capsule supplement twice per day (breakfast and dinner) in men with moderate or severe BPH symptoms [13]. The mean age was 55.8 years and the body mass index was 27.9 in the moderately symptomatic group (*n* = 10) and the mean age was 58.0 years old with a body mass index of 27.2 in the severely symptomatic group (*n* = 8) [13].

Equol as a nutraceutical supplement is currently available as a therapy to address BPH (and women’s menstrual) symptoms as complementary and alternative medicines (CAM), where some companies using a US Federal Drug Administration (FDA) dossier(s) evaluated equol as generally recognized as safe (GRAS) by self-affirmation based on data from oral or topical applications for over 5 years without side effects in more than 20,000 to over 50,000 human subjects, respectively (by application) [13,81,116,122,123].

**Figure 8:**
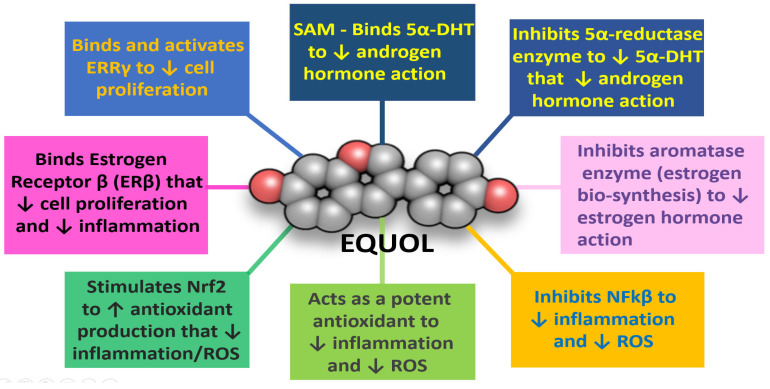
Equol’s Mechanisms of Action. Symbols/Abbreviations: SAM = selective androgen modulator by binding to free 5α-Dihydrotestosterone (5α-DHT); NFkβ = nuclear factor-kβ, a pro-inflammatory transcription factor; Nrf2 = nuclear factor erythroid 2-related factor, a transcription factor that regulates the production of antioxidants and detoxifying enzyme to combat oxidative stress; ROS = reactive oxygen species, a key signaling molecule that plays an important role in the progression of inflammation; ERRγ = Estrogen-related receptor gamma is an orphan nuclear receptor that has been shown to suppress cell proliferation and tumor growth of androgen-sensitive and androgen-insensitive prostate cancer cells.

Equol’s mechanisms of action: (1) directly by binding free serum 5α-DHT [13,119], (2) as a 5α-reductase enzyme inhibitor [119], (3) as a moderate aromatase enzyme inhibitor [114,115], (4) by inhibiting NFkβ and stimulating nuclear factor-erythroid factor 2-related factor 2 (Nrf2) expression to decrease inflammation and enhance antioxidant/detoxification enzyme expression, respectively, (5) as a SERM to protect the prostate by binding ERβ [13,113], (6) by binding estrogen-related-receptor-gamma (ERR-γ) that appears to play an important role in prostate health [13,118], and (7) for approximately 6 to 8 h (T ½ half-life) after taken orally [109] without side effects; then equol may provide beneficial efficacy to treat BPH symptoms that are not present in current pharmaceutical treatments (see Figure 8).

Finally, the gut microbiota-assisted synthesis, cellular interactions and synergistic aspects of equol as a potent anticancer isoflavone has been reported [124]. In summary, one recent review covered “equol producers”, which are a subset of certain populations have the ability to convert daidzein into the more potent equol compound under the action of the gut microflora that exerts strong and multifaceted anticancer actions. These include apoptotic, cell cycle arrest mechanisms, antiestrogen and anticancer actions via ER subtypes, antioxidant and anti-inflammatory, and synergism with current anticancer agents such as tamoxifen [124].

### 4.2. Beta-Sitosterol (BST) and BPH

**Figure 9:**
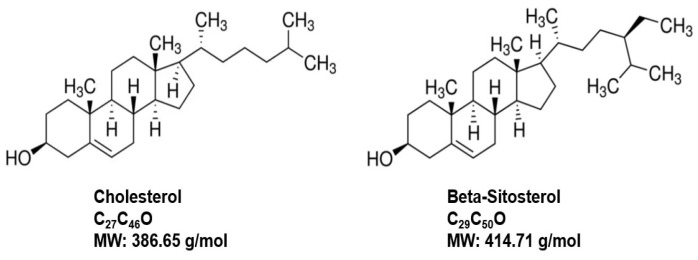
Comparison of the chemical structures, molecular formulas, and molecular weights between cholesterol and beta-sitosterol.

Phytosterol are biologically active compounds found naturally in plant cell membranes, with a chemical composition similar to animal cell membranes that are composed of cholesterol among the lipid-bilayer components [125,126,127] (Figure 9). Beta-sitosterol (BST), a white powdery organic substance, is one of the most abundant naturally occurring phytosterols in plants and is found in many food products such as cereals, fruits, nuts, vegetables, and vegetable oils, etc. [125,126,127]. BST has GRAS status in the US with applications in medicine, agriculture, and chemical industries [125,127]. A long history of research on BST from 1922 until today includes antioxidant, anxiolytic, lipid-lowering, antidiabetic, anti-inflammatory, anticancer and immunomodulatory, respiratory, wound healing and anti-viral protective effects among many other applications, in addition to its use for BPH, which have been reviewed elsewhere [125,126,127,128].

Early pharmacokinetic studies on BST showed low GI absorption at approximately 5–10%, a half-life (T ½) of around 3 h, and effective oral dosing ranging from 60 to 195 mg per day [129]. Additionally, in the late 1990s a multicentric, placebo-controlled, double-blind clinical trial (*n* = 177 men) reported by Klippel et al. examined BST (at 130 mg per day) for the treatment of BPH over a six-month interval [130]. IPSS values, peak urinary flow rates (Qmax), and post-void residual urinary volumes (PVR) were recorded pre-, during and post-treatment. The obtained results indicated significant improvements over placebo control levels, increased Qmax (4.5 mL/s) and decreased PVR values (33.5 mL) in the BST-treated men, which suggested that BST was an effective option for the treatment of BPH [130].

A more recent clinical study by Cosentino et al., 2018, examined the efficacy and safety of a combination dietary supplement containing curcumin, beta-sitosterol, and proanthocyanidins, in 100 men with LUTS caused by BPH [131]. Dosing was one tablet per day for 3 months, and the measured parameters included IPSS values and urinary flow levels. The study results suggested that the combination dietary supplement therapy was effective for almost all the IPSS parameters qualified with significantly increased urinary flow rates by a majority of the enrolled subjects without adverse side effects [131].

Sudeep et al., 2019, compared the efficacy of BST enriched saw palmetto (VISPO) versus conventional saw palmetto (SPO) at 200 or 400 mg/Kg body weight administrated orally daily for 28 days in alleviating BPH parameters using a testosterone-induced BPH rat model [132]. The measurement parameters included serum testosterone levels and prostate tissue analysis (histology plus inflammatory and apoptotic biomarkers via Western blot assays). In general, the obtained data showed experimental evidence that BST-enriched saw palmetto (VISPO) was more efficacious compared to the saw palmetto treatment (SPO) only (versus control animal values), which decreased prostate hyperplasia, testosterone levels, and for alterations in the expression of inflammatory and apoptotic biomarkers [132].

In a follow-up clinical double-blind, placebo-controlled randomized comparative investigation, the same authors, Sudeep et al., 2020, examined the efficacy and safety of a BST-enriched saw palmetto (VISPO) (3% BST) versus conventional saw palmetto (SPO) (0.2% BST) in adult men (ages 40–65 years of age, *n* = 33 per treatment group) with symptomatic BPH for 12 weeks [133]. Dosing was twice per day of capsules (containing 200 mg of extract—active ingredient), while placebo capsules contained maltodextrin for 12 weeks. The measured parameters included IPSS values, quantified urine flow (Qmax), 5α-reductase enzyme activity, prostate-specific antigen (PSA) analysis, and the Aging Male Symptoms (AMS) scale and Androgen Deficiency in Aging Male (ADAM) questionnaires. The overall results demonstrated that BST-enriched saw palmetto (VISPO) was significantly superior to conventional saw palmetto (SPO) for a majority of the quantified parameters that were evaluated, except that 5α-reductase enzyme activity was not significantly reduced [133]. This study claims to provide the first clinical evidence of improved efficacy of saw palmetto due to the enriched content of BST to improve BPH symptoms in men via the synergistic effects of the functional active ingredients in this dietary supplement [133].

Lastly, the molecular mechanisms of BST in preventing cell proliferation via pro-apoptotic, anti-metastatic, anti-invasive, and chemo-sensitizing actions that include factors such as p53 protein, B cell lymphoma-2 (Bcl-2), ROS, cell cycle blockade, interleukins, vascular endothelial growth factor (VEGF), mitogen activated protein kinase (MAPK), and PCNA pathways with applications to prostate disorders, but tumor progression, in general, have been recently reviewed [134].

### 4.3. Pumpkin Seed Extract and BPH

Pumpkin (*Cucuribita pepo*) is an herbaceous vine, originally native to America, now grown worldwide and has high nutritional and protein value (where lipids comprise 50% and protein 30% of the seeds) [135,136]. Pumpkin seeds contain, in addition to protein and fatty acids, vitamins [folates, niacin (B3), vitamins A, C, and especially E], minerals (zinc, phosphorus, manganese, potassium, magnesium, copper and iron) plus phyto-nutrients such as β-carotene, β-crypto-xanthin, lutein, and zeaxanthin) [135,136,137] (Table 3). Notably, the high zinc levels in pumpkin seeds are thought to ameliorate BPH symptoms by providing optimal levels of this important mineral along with its 5α-reductase enzyme inhibiting actions via the phyto-nutrients such as the phytosterols [137,138,139]. For example, a daily intake of zinc is required, because the body has no way to store it long term [140]. Recall, zinc is an essential trace mineral involved in many beneficial physiological functions, but pertaining to BPH it protects against oxidative stress and assists with DNA repair [140].

Pumpkin seeds have several of the same attributes as β-sitosterol (antioxidant, anti-inflammatory, cholesterol/blood pressure/diabetic control, etc.) [135,136,137]. Notably, while pumpkin seeds have been used traditionally for a variety of therapeutic applications one of the most frequent uses, both traditional and modern, continues to be for BPH [135,136,137]. The nutritional supplement doses for pumpkin seeds range from 100 to 1000 mg per day. This section summarizes the most recent findings involving pumpkin seed (extracts in most cases) for addressing BPH symptoms.

In 2021, Kang et al. reported using a rat model that pumpkin seed oil ameliorated BPH parameters by lowering prostate 5α-reductase enzyme expression, which confirmed previous results demonstrating this benefit [139].

In 2019, Leibbrand et al. used a single-arm, monocenter pilot study of 60 men with symptoms of BPH (IPSS of 14.8), that investigated the effects of a proprietary oil-free hydroethanolic pumpkin seed extract [141]. Subjects ingested oil-free hydroethanolic pumpkin seed extract (500 mg) once daily (before going to bed) for 3 months. After 12 weeks of supplementation, a significant symptom reduction from baseline (average, 30.1%) was observed based on total IPSS. Symptom alleviation also had a high impact on QOL and was significant after 8 and 12 weeks of intervention. Nocturia significantly decreased over time, as confirmed by IPSS questionnaire and bladder diary. Postvoid residual urine volume was significantly reduced at the end of intervention (from 83.67 mL at baseline to 63.11 mL after 12 weeks). In general, the results indicate that the oil-free hydroethanolic pumpkin seed extract was well tolerated (without adverse effects) and provided significant health benefits for BPH-related symptoms [141].

In 2021, Zerafatjou et al. investigated the effects of a pumpkin seed (720 mg) oil versus tamsulosin (0.4 mg) for BPH symptom relief in a single-blind randomized clinical trial for 3 months [142] with a total of 73 men (mean age 63.6 years old; weight, height and BMI were similar), 34 in the tamsulosin and 39 in the pumpkin seed group. There was a significant decrease in IPSS and significant increase in QOL values for both treatment groups, but the changes from baseline to 1 and at 3 months was significantly greater in the tamsulosin versus the pumpkin seed group. None of the pumpkin seed-treated subjects experienced any adverse effects, whereas dizziness (6%), headache (3%) and retrograde ejaculation (3%), and erythema with pruritus were observed in the tamsulosin group. The overall outcome of this study was that pumpkin seed treatment decreased BPH symptoms without adverse effects while tamsulosin appeared to be more effective (than the pumpkin seed treatment), but with side effects [142], suggesting that men may select natural therapeutic nutritional supplementation over pharmaceutical drug treatments based upon recent surveys [21,22].

In 2022, Theil et al. in a 24-month clinical study of 83 men (mean age 65.2 ± 8.7 years) with moderate LUTS/BPH symptoms, showed that pumpkin seed extract (500 mg capsules) taken twice per day significantly improved IPSS levels after 12 and 24 months in over 50% of the subjects [143]. The proportion of patients with QOL scores at baseline (mostly satisfied) was 11 percent that rose to 62 percent and 73 percent at 12 and 24 months, respectively. No adverse events were reported, and the AMS and International Index of Erectile Function (HEF-5) scores indicated no negative impact on function during the treatment interval [143]. The authors concluded that pumpkin seed extract provided significant relief in men with moderate LUTS/BPH symptoms without side effects.

Vahlensieck et al., in 2022, in a meta-analysis of two randomized placebo-controlled trials over 12-month evaluated the beneficial effects of pumpkin seed extract on LUTS and quality of life in men with BPH [144]. Specifically, the safety and efficacy of a well-tolerated proprietary pumpkin seed soft extract (PSE) was investigated in the Bach [145] and GRANU studies [146]. Both trials examined LUTS/BPH patients with IPSS > 13 at baseline. The original Bach study demonstrated positive effects of PSE compared to placebo, but no difference between treatments was observed in the GRANU study. In the combined studies, 687 subjects were in the PSE group, while 702 patients were in the placebo-control group [144,145,146]. For the variables study, baseline IPSS, and center size had a relevant influence on treatment response. Vahlensieck et al. concluded that although the Bach and the GRANU study showed contradictory results, the analysis in a pooled analysis pointed towards an advantage of PSE; namely, more patients in the PSE group showed an IPSS improvement of at least 5 points after 12 months [144,146]. Therefore, the results of this meta-analysis suggest that patients with moderate LUTS/BPH may benefit from PSE treatment in terms of symptomatic relief.

Finally, in 2020, Nemer et al. examined a combination treatment of pumpkin seed, isoflavonoids, and cranberry mix in LUTS/BPH subjects in a multicenter open-label one-arm pilot study [147]. Male patients were recruited, who still had LUTS/BPH symptoms despite current use of alpha-1 blockers, which included 128 men (61.8 + 9.9 years of age). The treatment mix tablet contained 550 mg of pumpkin seed extract, 50 mg of soy isoflavones and 50 mg of cranberry taken twice per day for 90 days, and IPSS, QOL, and HEF-5 were scored at baseline, 30, and 90 days. IPSS values significantly decreased from baseline from a value of fifteen compared to 30 days of a score of eleven to 90 days where the IPSS score was nine. QOL values significantly increased from baseline, while HEF-5 levels also improved [147]. The results suggested that the combination treatment of pumpkin seed extract, soy isoflavones and cranberry relieved LUTS/BPH symptoms in men and had a mild beneficial effect on erectile function. Remarkably, the obtained results were in men who were on alpha-1 blockers that did not previously alleviate their LUTS/BPH symptoms.

### 4.4. Saw Palmetto and BPH

Saw palmetto has a long historical record starting in 1575 [148]. Native Americans (in the Florida region) were known to use the fruit of saw palmetto (*Serenoa repens*) from the American dwarf palm tree (3 to 9 feet tall) to treat urinary and reproductive disorders [148]. In April 1877, Read and Abraham A. Solomons, manufacturing pharmacists in Savannah, introduced saw palmetto to the medical profession at the second annual meeting of the Georgia Pharmaceutical Association held in Atlanta, Georgia [148]. Interest in saw palmetto products increased in the early 1990s, as more scientific evidence of their safety and efficacy was published, particularly in Germany, where federal regulations at the time required a high level of scientific evidence for phytomedicinal products, which already had been on the market for decades. Since 2000, consumer interest in saw palmetto products has continued to grow in the United States, and saw palmetto was the 13th top-selling herbal dietary supplement ingredient in natural retail outlets in 2020 [148,149]. Since then, saw palmetto extract (SPE) has been commonly used to treat BPH. It has been used in Europe for decades, while increased use in the US over the past 30 years came about because of side effects from pharmaceuticals that treat BPH and the numerous scientific reports which provided evidence for saw palmetto’s safety and efficacy in treating LUTS and BPH-related symptoms [148,149,150,151,152].

There are three main types of saw palmetto extracts: hexanic, supercritical CO_2_ and ethanolic, with each having different phytochemical compositions [151,152]. However, the one most tested for efficacy and safety has been the hexanic *S. repens* extract (HSE) [149,150,151]. Saw palmetto in US supplement products (liquid) contain more than 90 percent fatty acids (laurate, myristic, palmitate, oleate, and linoleate acids), and low levels of phytosterols (campesterol, stigmasterol and β-sitosterol [149,150,151,152,153] (Table 4 and Table 5).

Notably, the HSE was first approved in Europe for BPH in 1981 [154]. The recommended dosage at 320 mg once daily or 160 mg twice daily, administered orally [149,150,151,152], where long-term use has been reported as well tolerated. However, in some long-term studies the low frequency adverse events reported included headache and abdominal pain, but without the adverse sexual effects observed with pharmaceutical alpha-1 blockers or 5RIs [154,155].

The mechanisms of action of HSE include (1) anti-androgen by non-selective inhibition of both type I and type II isoenzymes of 5α-reductase versus finasteride, which selectively inhibits only the type II 5α-reductase enzyme [149], (2) pro-apoptotic by inhibition of the growth of the prostate via blockade of insulin-like growth factor-1, plus the increased B-cell lymphoma-associated X (Bax)/Bcl2 ratio observed in prostate tissue sample from subjects (via oral dosing of HSE at 320 mg daily for 3 months compared to untreated control values) [149,156], and (3) anti-inflammatory actions by significantly reduced expression of inflammation-regulated genes including IL-6, CCL-5, CCL-2, COX-2, and iNos plus the downregulation of other pro-inflammatory factors [149,150,151,152]. Finally, it has been suggested that HSE may relax lower urinary tract smooth muscle by the inhibition of alpha-1 adrenergic and muscarinic receptors [149].

Active ingredients in HSE include the following: (1) fatty acids (lauric and myristic acid) have been shown to inhibit both 5α-reductase type I and type II enzymes, while olieic acid and linolenic acid inhibit the type I enzyme, plus several fatty acids showed inhibition of alpha-1 adrenergic receptors that may relax smooth muscle contraction in the prostate [149,152,156] and (2) phytosterols, which are relatively low in HSE but may contribute to reduced prostatic inflammation [149,150,151,152].

Over the past 40 years, there have been many reports of the chemistry, pharmacology, and clinical studies on saw palmetto, HSE and other aspects of this phytochemical product which have been reported in several reviews and meta-analyses [103,148,151,153,154]. However, in brief, the reports from the past 5 years are summarized herein.

Vela-Navarrete et al. in 2018 examined the efficacy and safety of HSE (Permixon^®^, Castres, France) for the treatment of LUTS/BPH by conducting a systematic review and meta-analysis of randomized controlled trials and observational investigations that covered 27 studies with a total of 447 patients [150]. The authors concluded that HSE (Permixon^®^) (at 320 mg per day or 160 mg taken twice per day over weeks to months) significantly reduced nocturia and improved Qmax compared to placebo control values and had similar efficacy to tamsulosin and (short-term use of) 5α-reductase inhibitors in relieving LUTS/BPH. The HSE (Permixon^®^) appeared to be an efficacious and well-tolerated therapeutic option for the long-term medical treatment of LUTS/BPH [150].

Kwon in 2019, and Csikos et al. in 2021, reviewed the use of HSE for BPH, and reported the benefits of the fatty acid and phytosterol components including the mechanisms of actions such as the anti-androgenic, pro-apoptotic and anti-inflammatory effects from several studies [103,150]. All the authors cited randomized clinical trials about the efficacy of HSE. However, Kwon pointed out that the variability of therapeutic efficacy of the HSE (formulas) may be due, in part, to the lack of standardization of HSE (formulations) [149]. On this point, the work of Penugonda and Lindshield (2013) showed variability of saw palmetto (active ingredients) in seven different nutraceutical supplements sold in the U.S. [153].

In 2022, Blair reviewed HSE (Permixon^®^) for the symptomatic relief of BPH symptoms by examining 21 different studies (representing 361 patients) that, in some cases, specifically compared Permixon^®^ to alpha-1 blockers or 5α-reductase inhibitors [152]. The rationale for this review was to address men wishing to avoid the side effects of alpha-1 blockers and/or 5α-reductase inhibitors, where HSE Permixon^®^ treatment avoided these adverse events especially those related to sexual function. The analysis indicated that the efficacy of HSE was similar to that of an alpha-1 blocker in terms of improving voiding and storage symptoms, increasing urinary flow, and reducing prostate inflammation and volume [152]. HSE Permixon^®^ was also as effective as 5α-reductase inhibitors and/or alpha-1 blockers at improving LUTS/BPH parameters and QOL. In general, HSE was well tolerated and offered a therapeutic option for the treatment of BPH [152].

In 2022, De Nunzio et al. reported on the combination therapy of alpha-1 blockers with HSE versus monotherapy in the treatment of LUTS/BPH from five different clinical studies (mean age ranged from 55.3 to 68.3, representing 1292 subjects with HSE dosing at 320 mg/day and alpha-1 blocker(s) dosing 0.2 to 0.4 mg/day from 6 to 13.5 months) [157]. The authors concluded that HSE in combination with alpha-1 blockers provided greater symptom relief and fewer adverse events (sexual dysfunction) in patients with LUTS/BPH than with alpha-1 blockers alone. The implications were that combination therapy of HSE and alpha-1 blockers for moderate LUTS/BPH subjects might be an effective strategy for preserving their sexual function, especially having higher BMI and/or metabolic syndrome disorders [157].

Finally, Nickel et al. in 2022, in a major review/evaluation of the available scientific evidence (safety and efficacy) of HSE for LUTS/BPH treatment using national and international urological guidelines by an international panel of urology experts generated consensus statements, which supported HSE use [158]. Specifically, the general overall conclusion was that HSE should be considered as a treatment option for men with mild-to-moderate LUTS/BPH symptoms as an alternative to watchful waiting [158].

### 4.5. Lycopene and BPH

**Figure 10:**
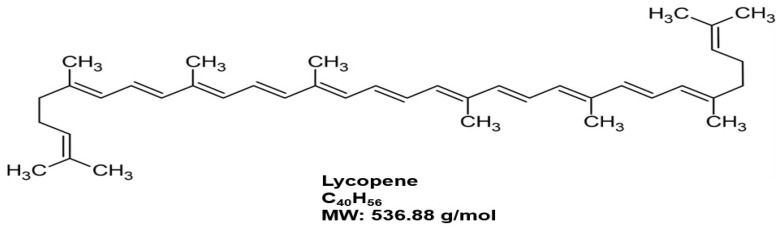
The chemical structure, molecular formula, and molecular weight of Lycopene.

A natural antioxidant, lycopene contained in plants has gained importance in preventing oxidation of fats/oils and foods, also as a food additive, along with its many human health benefits [159,160]. Lycopene is a lipophilic carotenoid hydrocarbon pigment found in red, pink, and orange fruit and vegetables such as tomatoes, apricots, melons, papayas, grapes, peaches, watermelons, and cranberries [159,160]. However, tomatoes are the most studied due to lycopene’s abundance in this fruit [159,160]. In 1876, Millardet, first discovered this compound (in tomatoes), and it was later named lycopene by Schunck in 1903 [160]. Lycopene is the most abundant carotenoid found in human serum and has been recognized as the most effective antioxidant among all the carotenoids [160]. Lycopene has 11 conjugated double bonds in its structure (Figure 10). The extended conjugated double bond system of these compounds is an important feature in the carotenoids responsible for their attractive colors because it forms a light absorbing chromophore [159,160].

Lycopene’s mechanism(s) of actions include (1) antioxidant characteristics by serving as a singlet oxygen and peroxyl radical scavenger, plus the combinations of lycopene with other antioxidants such as vitamin C and E express high scavenging activity levels to combat free radicals, and hence, oxidative stress, and also protect against DNA damage in both normal or cancerous human cells and lymphocytes [159,160,161]; (2) anti-inflammatory properties that result from its lipophilic nature, which has a close association with the cell membrane and enables it to regulate the inflammatory mediator signaling pathways and activate the expression of antioxidant genes (such as preventing the production of different types of cytokines (such as IL1, IL6, IL8, and TNF-α), chemokines, nitric oxide (NO), and cyclooxygenase that modulate the immune system response; it also inhibits the Nfkβ signaling pathway [159,160,161], and (3) anti-proliferation properties, where lycopene alters IFG-1 to decrease cellular proliferation. However, its stability is a critical factor for its functional aspects. Physical and chemical factors such as elevated temperature, exposure to oxygen and light, metallic ions (e.g., Cu^2+^ and Fe^3+^), extremes in pH, and active surfaces affect its stability [160].

From recent clinical studies on lycopene, Li et al. in 2019 examined the efficacy and safety of lycopene in 127 patients with moderate to severe LUTS/BPH by IPSS classifications [162]. Men were treated with oral lycopene (500 mg twice per day) for 16 weeks. At 8 and 16 weeks, they compared IPSS and QOL scores, prostate volume, PSA levels, Qmax, PVR, and incidence of adverse effects before and after treatment. The results showed that all the quantified parameters were significantly altered for the improvement of LUTS/BPH symptoms, except there was no significant difference in prostate volume before versus after treatment, and no adverse effects were reported by any of the patients [162].

Cicero et al. in 2019, and Kutwin et al. in 2022 published comprehensive reviews on lycopene as a dietary supplement for the treatment of LUTS/BPH [119,163]. In brief, oral lycopene at doses ranging from 10 mg to 500 mg, usually taken twice per day from 2 weeks to up to 6 months, in general, significantly improved LUTS/BPH symptoms (covering over 1000 subjects within an age range of 45–80 years) without adverse effects. The patients were classified as having moderate or severe LUTS/BPH symptoms according to IPSS values before lycopene treatment was initiated [119,163]. Furthermore, other comprehensive reviews on the health benefits of lycopene have been reported elsewhere [159,160,161].

In 2022, Carrasco et al. conducted a pilot study of 20 men (10 healthy versus 10 with moderate BPH symptoms via IPSS), mean age was 50 years old, both groups consumed lycopene (at 8 mg twice per day) for 30 days [164]. The measured outcomes included sleep quality, PSA, C reactive protein (serum), and total antioxidant levels (in urine). The obtained results showed improvement in IPSS values, PSA, and total antioxidant levels (in urine) in both the treatment groups, but especially in men with moderate BPH symptoms sleep quality significantly increased, and nocturia significantly decreased [164].

Combination phytochemical therapy was investigated by Morgia et al. in 2018 in a phase IV clinical study that compared HSE (320 mg) + selenium (50 mcg) + lycopene (5 mg) versus the phosphodiesterase-5 inhibitor (tadalafil at 5 mg) taken orally once per day for 6 months for the treatment of LUTS in 404 men (50 to 80 years of age) in a multi-center randomized controlled study [165]. The parameter outcomes included IPSS, Qmax and PVR, where both treatments significantly improved IPSS values (including the QOL scores), but were not significantly different from each other. Qmax values were significantly improved in the combination therapy and the phosphodiesterase-5 inhibitor treatment. For adverse events, four out of 101 patients (or 1 percent) in the combination therapy and 10 out of 120 subjects (or 8%) in the tadalafil treatment reported side effects. The authors concluded that the combination therapy of HSE + selenium + lycopene was equivalent to tadalafil treatment for improvements in the IPSS and Qmax parameters [165].

In 2021, Cormio et al. conducted a phase II prospective, randomized, double-blinded, placebo-controlled study aimed at determining the efficacy and safety of the novel whole tomato-based food supplement in reducing LUTS of patients with histologically validated BPH (via PBX) [166,167]. The food supplement combination therapy was studied in 40 men (mean age 65 years old) that contained carotenoids (500 mg), lycopene (190 mg) and flavonoids (200 mg) as the main active ingredients in a 5 g sachet taken once per day for 2 months. Compared to placebo control values, there was a trend in the combination therapy treatment men for reduction in PSA levels, while there were significant improvements in IPSS and QOL scores at the end of the study [166]. The authors concluded that the tomato-based food supplement represented an efficacious option for the treatment of symptomatic BPH compared to pharmaceutical treatments, since this food supplement was free of side effects and highly accepted among the patients tested [166].

Finally, the anti-inflammatory and healing properties of oral lycopene along with dutasteride treatment on reducing transurethral resection of the prostate (TURP) bleeding were examined by Nugroho et al. in 2019 [168]. Twenty-two patients diagnosed with BPH were randomly assigned to two groups (*n* = 11 each). Thirty days prior to TURP, men in the control group were given daily oral dutasteride 0.5 mg and a placebo pill and subjects in the treatment group were given dutasteride 0.5 mg and lycopene 30 mg daily. For this study, the parameters measured were pre- and post-TURP plasma erythrocyte count and micro-vessel density (MVD) of resected prostate tissue stained for histology evaluation. The mean MVD in the control group was significantly higher versus the intervention group (28.2 ± 12.3 vs. 18.3 ± 7.6 vessel/mm^2^) and reduction of post-TURP plasma erythrocytes was significantly higher in the control group compared to the intervention group (−0.34 ± 0.18 vs. −0.17 ± 0.12). The results suggested that daily consumption of dutasteride and lycopene for at least 30 days reduced the formation of blood vessels in the prostate and reduced blood loss in post-TURP examinations [168].

### 4.6. Stinging Nettle and BPH

Nettle (*Urtica dioica*) is a perennial herb native to Europe, Asia, North Africa, and North America that grows to a height of 3 to 7 feet [169,170]. It is often referred to as “Stinging Nettle” because it has “trichomes”, or hollow hairs, on its leaves and stem, which act like needles that inject histamine, formic acid, and other chemicals that produce a stinging sensation [169,170]. Nettle, unlike many other plants, tends to produce only male or female flowers throughout each plant, thus giving it the name dioica, meaning “two houses” [169,170,171]. Nettle use has been recorded as far back as the Bronze Age (3000 BCE—1200 BCE), and there are records (59–45 BCE) where Julius Caesar’s troops rubbed it on their limbs to help them stay awake and alert during the night in battle [169,170,171]. In addition to its use in herbal therapies, nettle also has been used as a textile (similar to flax) in the 1500–1600s in Scotland and during WWI and WWII as a substitute for cotton. There are many human health applications of stinging nettle (food, cosmetic, etc., which have been reviewed elsewhere [169,170,171,172,173,174], including its use in enhancing farmed fish immunity to be more resistant against bacterial infections [173,174]. However, the most recognized human health supplement application of stinging nettle (root) is for the treatment of BPH [119,175,176,177,178,179].

Stinging nettle can be extracted from fresh herbal plants by industrial aqueous, alcoholic-organic and/or hydroalcoholic methods, and distillation processes, where good yields can be 0.74 L of hydrolate from 1 Kg of starting material [172,179]. The nutritional composition and various compounds in stinging nettle have been identified (Table 6, below), including protein, dietary fiber, flavonoids, phenolic acids, carotenoids, organic acids, and fatty acids [173,175].

Stinging Nettle’s mechanism(s) of actions include (1) antioxidant characteristics by serving as a superoxide scavenging agent, and/or stimulating the increase in catalase (CAT), superoxide dismutase (SOD), and glutathione along with protecting against lipid peroxidation, and DNA damage [172,173,174], (2) anti-inflammatory properties by inhibiting COX-1 and COX-2, NFkβ signaling pathway, IL-1 and IL-2, interferon (INF), and TNF-α2 [172,173,174], (3) anti-proliferation properties, where stinging nettle inhibits not only the binding of androgens to their transporter proteins [i.e., sex hormone binding globulin (SHBG)], but also their binding to prostate membrane receptors that contribute to the proliferative effects on prostate tissues along with inhibiting the aromatase enzyme (for estrogen biosynthesis), thus altering prostate growth [172,173,174] and, (4) anti-bacterial/viral activities by inhibiting Gram-negative and Gram-positive bacterium and viruses (respiratory syncytial and cytomegalovirus) by its component compounds of alkaloids, flavonoids, phenols, and saponins [173,174,180,181,182].

One of the largest randomized, double-blind, placebo-controlled clinical studies to examine the effects of stinging nettle was reported in 2005, where 558 patients (55 to 72 years of age) were administrated 120 mg of *U. dioica* root extract for 6 months [183]. The obtained results showed that 81 percent of the men displayed significant improvement in LUTS/BPH symptoms, Qmax, and QOL scores [183]. Additionally, PVR levels were significantly decreased with a modest decrease in prostate size, but no alterations in PSA or testosterone levels were observed with stinging nettle oral supplementation [183]. Since 2005, other clinical studies have been performed that are summarized below (including combination therapies), and general reports/reviews are available elsewhere [119,169,170,171,172,173,174,175,176,177,182].

In 2020, Karami et al. reported a randomized controlled trial investigating the efficacy of stinging nettle root extract in 30 LUTS/BPH patients (administered 450 mg tablet extract per day for 12 weeks) and in 30 men that served as controls (placebo group) [184]. The measured parameters were IPSS, C-reactive protein levels, malondialdehyde (MDA), and SOD activities. The results revealed immediate effects (within days) in improving IPSS values, a small effect on C-reactive protein, intermediate effects on MDA and SOD activities, without any adverse effects after 3 months of stinging nettle on the oral supplementation treatment [184].

Ibishev et al. in 2019 reported a comparative study investigating HSE alone (at 320 mg taken once per day) versus HSE (at 160 mg) in combination with stinging nettle (at 120 mg) taken twice per day for 3 months in 51 men per treatment group (age ranged from 48 to 64 years) to determine of efficacy of each treatment [185]. The results showed a significant improvement in LUTS/BPH parameters via IPSS values, significant increase in Qmax with a significant decrease in PVR in both treatment groups. However, when group comparisons were made the combination treatment displayed better results compared to HSE therapy alone for addressing LUTS/BPH symptoms in men [185].

Kirscher-Hermans et al., in 2019, reviewed four randomized, placebo-controlled clinical trials that examined the efficacy of a promising herbal preparation (called WS PRO 160/120 available in Germany) for the treatment of patients with LUTS/BPH related symptoms [186]. This herbal supplement contains 160 mg of HSE plus 120 mg of stinging nettle (root) extracts, and the four reviewed studies represented 980 patients (mean age ranged from 65 to 68 years) that were administered one capsule twice per day for 24 to 60 weeks. In all the trials, significant symptomatic improvement was recorded via IPSS, QOL and Qmax values while PVR was significantly reduced compared to placebo parameters and comparable with the 5α-reductase inhibitor (finasteride) or the alpha-1 blocker, tamsulosin. The adverse events were few with the herbal combination treatment compared to the tolerability and safety profiles of the reference drugs (5RI or alpha-1 blocker), which were included in two out of the four studies. The authors concluded that the combination HSE and stinging nettle extract treatment was a valid alternative in the treatment of patients with moderate BPH, especially with the view to sexual function and good quality of life especially for long-term use [186].

Finally, an in vitro study by Saponaro et al. in 2020 evaluated the antioxidant and anti-inflammatory activity of a combined formulation of HSE and stinging nettle extract using a human model of BPH (i.e., BPH-1 cells) [187]. The results confirmed both the antioxidant and anti-inflammatory effects of the combination treatment by a reduction in ROS, IL-6 and IL-8 production and a reduction in NFkβ translocation inside the nucleus using the human androgen-independent prostate model, PC3 cells. The authors concluded that the combination treatment of HSE and stinging nettle extract supported the hypothesis that ROS reduction is directly implicated in the decrease of the NFkβ pathway to cause reduced expression of inflammatory cytokines. Consequently, the reduced inflammation contributed to the mitigation of prostate enlargement and BPH symptoms [187].

### 4.7. Green Tea

Green tea has an interesting history. It originated in China around 3000 BCE, where a later written account described the discovery that occurred accidently when the Chinese Emperor Shennong mistakenly drank water with a dead tea leaf boiled inside [188,189]. He found the flavor refreshing; thus, a new beverage was born. It was not available to the Chinese general public until the 14th century for liquid refreshment and medicinal purposes. Then, it spread to many different neighboring regions, and in the 19th century it traveled West with European explorers, where today its Great Britain’s national beverage (along with black tea) [188,189]. Today, green tea is one of the most popular drinks in the world and the least oxidized compared to other teas [190]. In the last 30 years, the popularity of green tea has greatly increased and has been extensively investigated for its many health benefit including its protective role against various types of human cancers [10,12,119,190,191,192,193]; however, this narrative overview is focused on BPH.

Green tea comes from the *Camellia sinenis* plant that is an evergreen shrub or small tree native to East Asia and Southeast Asia, where fresh leaves contain caffeine (around 4 percent), are a rich source of polyphenols and catechins along with theanine, vitamins (B2, C, E, and folic acid), saponins, GABA and minerals (potassium, calcium, phosphorus, etc.) [190,194]. The polyphenols in green tea are credited with beneficial properties against several diseases/disorders via biological and molecular mechanisms [190,195].

**Figure 11:**
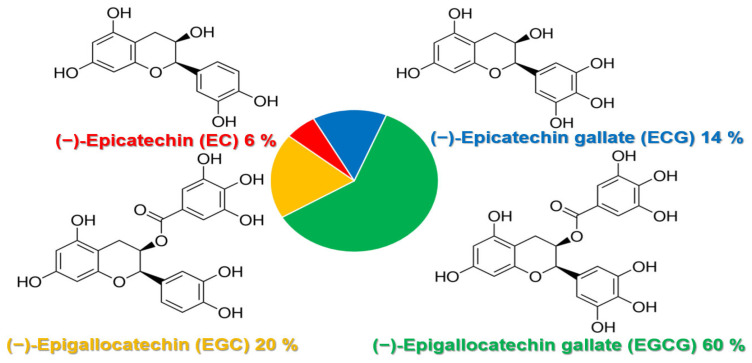
The Four Major Catechins in Green Tea (chemical structures, and approximate percent distribution). EGCG is the major catechin (by percentage in abundance, which accounts for most research carried out with green tea.

The distinctive polyphenolic compounds present in green tea are called catechins, which make up 80–90% of the flavonoids and about 40% of the water- soluble solids [190]. The four major catechins are (-)-epicatechin (EC), (-)-epigallocatechin (EGC), (-)-epicatechin gallate (ECG), and (-)-epigallocatechin gallate (EGCG) [190,195]. The distribution of the catechins varies; however, EGCG accounts for around 60%, followed by EGC at 20%, ECG at 14% and EC at 6% [190,195] (Figure 11). EGCG is the major catechin in green tea that accounts for most of the research conducted to date on various disorders and human cancers [192,193,195]. For example, one cup of green tea contains up to 200 mg of EGCG, although some reports suggest three to five cups of green tea per day provides a minimum of catechins per day [196]. The bioavailability of EGCG has been determined in adult health volunteers (men *n* = 5, women *n* = 5; mean age 30 years old) after a single dose of green tea extract supplement (approximately 200 mg) by pharmacokinetic studies where the Cmax (maximum concentration) was approximately 6 mg/mL/Kg in men and women, while the T ½ (half-life) was highest in men at 154 min vs. 117 min in women [196]. This recent report on the bioavailability of green tea catechins confirms previous pharmacokinetic studies in humans [195,197].

Notably, green tea has been extensively studied; however, the controversies regarding its benefits and risks still exist, but the numerous health-promoting benefits outweigh its few reported risk effects [197,198]. Nevertheless, this is a matter of perspective, since herbal dietary supplements may present potential hepatotoxicity, if consumption exceeds the recommended dosage [198]. Conversely, all clinical studies have reported that green tea in liquid or dietary supplements (at various doses) is well tolerated without side effects [10,119,190,192,193,194,195,196,197].

There are several mechanisms of action for the health benefits of green tea, since extensive investigations from in vitro, animal, and human results are available [10,11,12,103,119], but preclinical and clinical evidence of green tea has focused on the catechin, EGCG for almost all reported research reports [195,196,197,198,199]. Thus, EGCG will be the major coverage for the mechanisms of action involving green tea [103,105,106,119,190,191,195,196,197,198,199].

First, EGCG has been shown to induce apoptosis, arrest the cell cycle and inhibit cell proliferation (DNA replication) [103,105,119,199]. Flow cytometric analysis found a linear dose relationship between EGCG and the promotion of apoptosis via DNA fragmentation in LNCap and DU245 cell lines [105,111,199]. Furthermore, ECGC altered the transcription factors p53 and NFβ that lead to a change in the ration of Bax/Bcl-2 in a manner that favored apoptosis [10,111,199]. Additional studies showed that EGCG induced G0/G1 phase cell cycle arrest in both androgen-sensitive and androgen-insensitive cell lines in a dose-dependent manner [12,111,199]. EGCG also inhibits the expression of the highly conserved mini-chromosome maintenance (MCM) gene and protein (specifically MCM7) that are essential for genome replication, which is associated with the progression of cell proliferation and growth [195,199].

**Figure 12:**
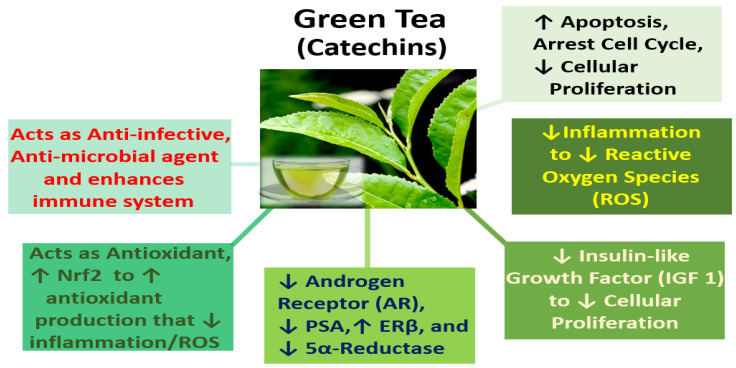
Green Tea (Catechins) Mechanisms of Action. Symbols/Abbreviations: PSA = Prostate-Specific Antigen; ERβ = Estrogen Receptor beta; 5α-Reductase = enzyme that converts testosterone to the most potent androgen 5-alpha dihydrotestosterone (5α-DHT); Nrf2 = nuclear factor erythroid 2-related factor, a transcription factor that regulates the production of antioxidants and detoxifying enzyme to combat oxidative stress; ROS = reactive oxygen species, a key signaling molecule that plays an important role in the progression of inflammation.

Additionally, EGCG is involved in regulating MAP kinases (P38/JNK/ERK), PI3K-Akt, and PKC to reduce cellular proliferation and growth [10,12,111,199]. For example, EGCG reduced the expression of ERK1 and 2 (MAP kinases) by 50–60% in vitro observed in DU-145 cells as well as other MAP kinases (p38, JNK) [10,12,111,199]. Additionally, green tea polyphenols decreased expression of PI3K (by 70%) and Akt (by 65%) in LNCap and DU-145 cell cultures [190,199]. Taken together, the results indicated that the green tea component compound, EGCG, induced apoptosis and arrested the cell cycle to decrease cell proliferation in prostate cells [111,195,199] (Figure 12).

Second, EGCG acts as an anti-inflammatory agent that targets the NFkβ and COX-2 inflammatory pathways, where EGCG was shown to decrease the DNA binding activity of NFkβ and reduced the expression of the p65 subunit of NFkβ in LNCap cells [190,191,195]. EGCG inhibited COX-2 as well as iNOS expression in vitro, which suggested an anti-inflammatory action in addition to inhibiting the NFkβ pathway [10,12,111]. Using a rat model, Zhou et al. in 2018 reported the ameliorative effects of EGCG against testosterone-induced benign prostatic hyperplasia and fibrosis [200]. The authors reported that the EGCG treatment improved prostatic hyperplasia and collagen deposition by attenuated prostatic oxidative stress and inflammation via reduced expression of AR, ERα, HIFα, TGF-β1, TGF-βR1 and p-Smad3, while enhancing the expression of ERβ in prostate tissue [200] (Figure 12).

Third, EGCG inhibited insulin-like growth factor and insulin-like growth factor binding proteins (IGFBPs) levels by 70 to 80% in prostate tissues of in vivo animal studies (TRAMP mice) [105,190,199]. A combination of green tea polyphenols (at 0.1% or 0.6%) in the drinking water of the mice displayed significant reductions in the insulin-like growth factor parameters compared to control values [199] (Figure 12).

Fourth, green tea and/or EGCG reduced the expression of androgen receptor (AR), prostate-specific antigen (PSA) levels and inhibited the 5α-reductase enzyme (responsible for 5α-DHT biosynthesis) in in vitro (cell culture) studies and in vivo using athymic nude mice [10,12,105,199]. In brief, in LNCap cells ECGC and/or green tea extract in concentrations of 10–20 µM significantly decreased AR gene and protein expression; in observations from in vivo data with nude mice, the administration of 0.1% green tea extract in the drinking water resulted in reduced prostate volume, and serum PSA levels [199]. However, in a systematic review and meta-analysis of randomized controlled studies, Sharifi-Zahabi et al., in 2021, reported that green tea had no significant effect on PSA levels [201]. However, the authors noted that this lack of support may be due to the heterogeneity among the studies analyzed, where larger sample sizes are required to make a definitive determination on the relationship between green tea’s effect on PSA levels [201]. In a cell-free assay, ECG and EGCG inhibited the 5α-reductase type I enzyme with an IC50 of 12 µM and 15 µM, respectively, which suggested decreased production of the potent androgen, 5α-DHT, in ameliorating BPH symptoms [199]. Lastly, Mitsunari et al. in 2021 reviewed in detail the changes in hormone-related molecules (androgens, estrogens, 5α-reductase) and eight different growth factors by green tea polyphenols [105] (Figure 12).

Fifth, EGCG acts as an antioxidant, stimulates Nrf2 (to increase antioxidant and detoxifying enzymes), decreases oxidative stress and enhances fertility [105,202,203,204,205]. Prior studies by Na and Surh, in 2008, showed that EGCG stimulated the master antioxidant and detoxifying signal Nrf2 that in turn induced expression of glutathione S-transferase, glutathione peroxidase, glutamate cysteine ligase, hemeoxygenase-1 (HO1), etc., to decrease oxidative stress [202]. Yao et al., in 2019, examined the effects of dietary EGCG on oxidative stress and the metabolism/toxicity of acetaminophen in the liver, where rats were fed diets with (0.5%) of EGCG or without EGCG supplementation for four weeks and were then injected intraperitoneally with acetaminophen (1 g/kg) [203]. The results showed that EGCG lowered hepatic oxidative stress and cytochrome P450 (CYP) 1A2, 2E1, and 3A, and UDP-glucurosyltransferase activities prior to acetaminophen injection. After acetaminophen challenge, the elevations in plasma alanine aminotransferase activity and histological changes in the liver were ameliorated by EGCG treatment. Thus, EGCG reduced cytochrome P450 (CYP)-mediated acetaminophen bioactivation, oxidative stress, and apoptosis, as well as increased autophagy and lower accumulation of toxic products in the liver [203]. Notably, in 2021, Mitsunari et al. reviewed in detail the changes in antioxidants, oxidative stress, and inflammatory-related molecules by green tea polyphenols [105]. Zhang et al., 2020, reported in a review the physiological activities of EGCG in improving fertility in humans and mammals due to its detoxifying and antioxidant effects, especially to reduce ROS [204]. In 2022, Beyaz et al. reviewed the antioxidant properties of EGCG that acts as a scavenger of free radicals, has redox characteristics, prevents the formation of reactive oxygen species, and has been shown to reduce cellular damage caused by oxidative stress [206] (Figure 12).

Sixth, the anti-infective and immune properties of EGCG have been studied for several years [207,208,209]. For example, Steinmann et al., in 2012, reviewed the actions of EGCG that bind to lipid membranes, which altered folic acid metabolism of bacteria and fungi by inhibiting the cytoplasmic enzyme dihydrofolate reductase [207]. In 2018, Reygaert reviewed the antimicrobial properties of the catechins that have been shown to be effective against several viruses, parasites, fungi, and even prions, but also noted that the anti-inflammatory and antioxidant effects of catechins may support/contribute to this mechanism of action [208]. Finally, Sun et al. in 2022 reported a comprehensive review on the effects of green tea and its components on the immune function, where the authors indicated that in immune-related diseases tea polyphenols are the most significant compounds that modify immune functions (of macrophages, natural killer cells, mast cells, basophils, and eosinophils, neutrophils, dendritic, T and B cells) for potential therapeutic applications in humans [209] (Figure 12).

Since green tea and its catechins have been extensively studied, almost all recent clinical investigations have examined prostate cancer rather than BPH [192,210,211]. However, presented herein are a few available clinical studies on green tea (catechins) covering BPH that have been published or are currently being conducted.

In 2014, Katz et al. examined the safety and efficacy of a green and black tea blend in 40 men (mean age 56 years old) with moderate to severe LUTS/BPH in a randomized, double-blind, placebo-controlled clinical trial [212]. In three treatment groups, men took capsules once per day for a total of (a) 500 mg tea blend (*n* = 15), (b) 1000 mg tea blend (*n* = 13) or (c) placebo-control (*n* = 12) for 12 weeks and the parameter outcomes included American Urologic Association symptom scores (AUAss), uroflowmetry (Qmean), PVR, C-reactive protein levels, a health survey (HS), and International Index of Erectile Function (IIEF) at 6 and 12 weeks of treatment. The results showed that oral administration of the green/black tea blend significantly improved LUTS/BPH symptoms (via the AUAss, Qmean urine flow and PVR values in the treatment groups versus placebo-controls) and the quality of life and physical function/sexual desire improved by the HS and IIEF scores in as little as 6 weeks, where the tea blend treatment was well tolerated without adverse effects [212].

In 2021, Mitsunari et al. reviewed the potential clinical usefulness of polyphenols (including green tea), where the authors concluded that no well-designed clinical trials with large populations have been conducted to determine the clinical use and safety in humans [105]. However, in 2020 a randomized double-blinded Phase II clinical trial began to evaluate the bioavailability, safety, effectiveness and validate the mechanism by which a standardized formulation of whole Green Tea Catechin, (Sunphenon^®^ 90D, Minneapolis, MN, USA) containing 405 mgs vs. placebo, administered for 24 months in a cohort of 135 men to manage prostate health progression (via active surveillance), which will conclude in 2024 [213].

While green tea and its catechins molecules have received much research attention over the past 20 years, further clinical studies are warranted to determine its safety and efficacy in treating LUTS/BPH in men.

Finally, other important aspects of green (and black) tea are the benefits of polyphenols on mediating gut microbiota where there is a two-way relationship that involves the metabolism and mechanism(s) of action to improve human health [214,215]. For example, ellagic acid is a polyphenolic compound present in green tea and other natural sources including pomegranate, strawberries, blackberries, raspberries, and walnuts, etc. [216]. Furthermore, upon ingestion of green tea or EGCG, microbial gut enzymes convert EGCG into gallic acid and epigallocatechin (EGC) [216,217]. Gallic acid can be subsequently degraded into urolithins and pyrogallol by decarboxylation (from gallic acid and ellagitannins) that were discovered almost 20 years ago in animal models and in humans [216,217]. In fact, urolithins (A and B) have been shown to accumulate in human breast, colon, and prostate tissue [216]. Previous studies have shown that urolithins disrupt the regulatory mechanisms in the G1 phase of the cell cycle upregulating apoptosis, control proliferation of epithelial cells, and decrease serum concentrations of prostate-specific antigen (PSA) as well as androgen receptor (AR) levels within prostate cells [216,217]. Moreover, the gut microbial metabolite pyrogallol is a more potent inducer of Nrf2-associated gene expression compared to its parent green tea compound EGCG [218], which suggests strong antioxidant and detoxifying effects. Recently, secondary polyphenol metabolites such as urolithins have been reported as anticancer compounds that can mediate cell cycle arrest, inhibit estrogen biosynthesis via the aromatase enzyme, induce apoptosis, promote autophagy, reduce oxidative stress, enhance antioxidant activities, and regulate growth factors [219].

## 5. Combination Nutraceutical Therapies for BPH

Just as combination therapies have been instituted for cancer treatments [220,221], more and more nutraceutical companies and academic/research investigations have advanced to combination nutraceutical active ingredients to provide the best efficacy without side effects. The apparent best viable option appears to be nutraceutical supplementation (via phytochemicals) and/or in combination with pharmaceutical treatments for LUTS/BPH. For example, in most of the previous sections for each of the natural phytochemicals covered there are examples of combinations of active ingredients that deliver multiple mechanisms of action and in most cases enhanced or synergistic effects were recorded along with pharmaceutical treatments (in some cases) to ameliorate the prostate disorders of LUTS/BPH. This advanced combination therapy is ongoing, which will lead to better and more effective ways to treat LUTS/BPH along with changes in diet and physical activity.

## 6. Conclusions

This narrative overview updates the factors that influence BPH in reference to diet, physical exercise, the prostate microbiome, and nutraceutical supplements such as vitamin A, C, D, and E (alone or in combination with) polyphenols and phytochemicals derived from plants that hold promise or are likely to alleviate LUTS/BPH symptoms (using reports and reviews published mainly in the last 5 years from January 2018 through January 2023). The risk factors for BPH include (a) age [60,65,66,69], (b) smoking (vaping) [69], (c) obesity/overweight [69,72], (d) ↑ insulin [53,57,61,69], (e) ↑ lipids [61,69,72], (f) diabetes [69], (g) hypertension [53,69], (h) depression [69], and (i) a “Western diet.” Conversely, vitamins A, C, D and E along with a “Mediterranean diet” or “Eastern diet” and polyphenols/phytochemicals nutraceutical supplementation with equol, β-sitosterol, pumpkin seed extract, saw palmetto, lycopene, stinging nettle, and green tea are associated with decreased BPH symptoms. Notably, urologists and medical associations are rethinking the role of plant-derived phytochemicals, because now they are considered as treatment options for men with mild-to-moderate LUTS/BPH symptoms as an alternative to watchful waiting [158]. While this perspective is evolving, further research is warranted on epigenetic factors such as diet, exercise, and nutraceutical supplementation to determine the importance and extent to which these therapies/remedies serve as innovations to ameliorate LUTS/BPH symptoms. This is especially the case where combinations of active ingredients (traditional drugs and/or multiple phytochemicals and vitamins) may be utilized to enhance the efficacy of treatments for LUTS/BPH.

## Figures and Tables

**Table 1:** The efficacy and safety of several common drugs for the treatment of BPH/LUTS [14,21].

**Alpha-1 Blockers: Mechanism of Acton—Relaxes Smooth Muscle in Prostate and Bladder**			
1. Tamsulosin (Flowmax)	**Dose**	**Adverse Reactions**	**Speed of Onset**
	0.4 mg	headache dizziness abnormal ejaculation runny/snuff nose confusion/depression	Days
2. Silodosin (Rapaflo)	8 mg	headache dizziness abnormal ejaculation	Days
**Non-Selective Alpha-1 Blockers: Mechanism of Action—relaxes smooth muscle in prostate and bladder**			
3. Alfuzosin (Uroxatral)	**Dose**	**Adverse Reactions**	**Speed of Onset**
	2.5 or 10 mg	dizziness upper respiratory tract infection	Days
4. Terazosin (Hytrin)	1 mg	dizziness asthenia postural hypotensive	Days
5. Doxazosin (Cardura)	1 mg	dizziness fatigue hypotensive	Days
**5 Alpha-Reductase Inhibitors: Mechanism of Action: inhibits the 5α-Reductase enzyme(s), types 1 and 2 for Dutasteride and type 1 for Finasteride**			
6. Dutasteride (Avodart)	**Dose**	**Adverse Reactions**	**Speed of Onset**
	0.5 mg	Impotence decreased libido ejaculation dysfunction	Months
7. Finasteride (Proscar)	5 mg	Impotence decreased libido breast enlargement	Months

**Table 2:** Overall IPSS values in Moderately or Severely Symptomatic Men over 4 Weeks Treatment of Equol. ▼ = Significantly decreased IPSS values compared to baseline levels.

4 Week Treatment Interval	Baseline	2 Weeks	4 Weeks
Moderately Symptomatic Men (*n* = 10)	13.9 (±0.7)	9.9 (±1.1) ▼	8.0 (± 1.1) ▼
Severely Symptomatic Men (*n* = 8)	26.1 (±1.4)	15.7 (±1.8) ▼	12.3 (± 1.7) ▼

In the severely symptomatic group of men the quality of life (QOL) score at baseline was 4.5 (mostly dissatisfied to unhappy) but improved to 2.8 (mostly satisfied) after 4 weeks of equol treatment.

**Table 3:** Nutritional Composition of pumpkin seed (nutrient value per 100 g). Source: United States Department of Agriculture (USDA) Nutrient Database. * Recommended Daily Allowance (RDA) not established (see below).

Principle	Nutrient Value	Percentage of RDA	Principle	Nutrient Value	Percentage of RDA
Energy	559 Kcal	28 %	**Electrolytes**		
Carbohydrates	10.71 g	8%	Sodium	7 mg	0.5%
Protein	30.23 g	54%	Potassium	809 mg	17 %
Total Fat	49.05 %	164%	**Minerals**		
Cholesterol	0 mg	0%	Calcium	46 mg	4.5%
Dietary Fiber	6 g	16%	Copper	1.343 mg	149%
**Vitamins**			Iron	8.82 mg	110%
Folates	58 µg	15%	Magnesium	592 mg	148%
Niacin	4.987 mg	31%	Manganese	4.543 mg	198%
Pantothenic acid	0.750 mg	15%	Phosphorus	1233 mg	176%
Pyridoxine	0.143 mg	11%	Selenium	9.4 µg	17%
Riboflavin	0.153 mg	12%	Zinc	7.81 mg	71%
Thiamin	0.273 mg	23%	**Phyto-nutrients**		
Vitamin A	16 IU	0.5%	Carotene-β	9 µg	*
Vitamin C	1.9 µg	3%	Crypto-xanthin-β	1 µg	*
Vitamin E	35.10 mg	237%	Lutein-Zeaxanthin	74 µg	*

**Table 4:** Fatty acid quantities (mg/gram) and composition (percentage of total fatty acids) in liquid saw palmetto supplement products sold in the United State. Mean of 7 different nutraceutical products (+ SEM).

Fatty Acids	mg/gram	Percentage
Laurate	170.8 (±18.0)	18.8 (±1.9)
Myristate	65.9 (±6.9)	7.2 (±0.7)
Palmitate	91.7 (±2.0)	10.1 (±0.2)
Stearate	22.6 (±1.4)	2.5 (±0.2)
Oleate	400.2 (±30.9)	43.8 (±3.2)
Linoleate	82.2 (±19.2)	9.3 (±2.4)
Total	908.5 (±9.6)	90.9 (±1.0)

**Table 5:** Phytosterol quantities (mg/gram) and composition (percentage of total phytosterols) in liquid saw palmetto supplement products sold in the United State. Mean of 7 different nutraceutical products (+ SEM).

Phytosterols	mg/gram	Percentage
Campesterol	0.28 (+0.04)	13.79 (+1.45)
Stigmasterol	0.14 (+0.02)	6.73 (+0.57)
β-Sitosterol	1.62 (+0.12)	79.48 (+1.99)
Total	2.01 (+0.15)	100.00 (+0.02)

**Table 6:** Nutritional Composition of stinging nettle (nutrient value per 100 g). Source: United States Department of Agriculture (USDA) Nutrient Database. * Recommended Daily Allowance (RDA) not established.

Principle	Nutrient Value	Percentage of RDA	Principle	Nutrient Value	Percentage of RDA
Energy	42 Kcal	2%	**Electrolytes**		
Carbohydrates	7.49 g	5.75%	Sodium	4 mg	< 1%
Protein	2.71 g	5%	Potassium	334 mg	7%
Total Fat	0.11 g	< 1%	**Minerals**		
Cholesterol	0 mg	0%	Calcium	481 mg	48%
Dietary Fiber	6.9 g	18%	Copper	0.076 mg	8.4%
**Vitamins**			Iron	1.64 mg	23%
Folates	14 µg	3.5%	Magnesium	57 mg	14%
Niacin	0.388 mg	2.4%	Manganese	0.779 mg	34%
Pyridoxine	0.103 mg	8%	Phosphorus	71 mg	10%
Riboflavin	0.16 mg	12%	Zinc	0.34 mg	3%
Thiamin	0.008 mg	<1%	**Phyto-nutrients**		
Vitamin A	2010 IU	67%	Carotene-β	1150 µg	*
Vitamin K	499 µg	416%	Carotene-α	114 µg	*
			Lutein-Zeaxanthin	4180 µg	*

## Data Availability

The data presented in this study are available on request from the corresponding author. Some data may not be publicly available due to funding sponsor intellectual property agreements.

## Abbreviations

α	alpha
β	beta
γ	gamma
µM	micromolar
Cmax	maximum concentration
dL	deciliter
g	gram
Kg	kilogram
L	liter
mcg	microgram
mg	milligram
mL	milliliter
mm^2^	square millimeter
n	number
ng	nanogram
nMol	nanomolar
T 1/2	half-life
5α-DHT	5alpha-dihydrotestosterone
5RI	5alpha-reductase inhibitor
ADAM	androgen deficiency in aging males
AKT	serine/threonine protein kinase
AMS	aging male symptoms
AP1	activator protein 1
AR	androgen receptor
AUAss	American Urologic Association symptom score
B3	niacin
Bax	B-cell lymphoma-associated X
Bcl-2	B cell lymphoma-2
BMI	body mass index
BMP	bone morphogenetic protein
BST	beta-sitosterol
BPH	benign prostatic hyperplasia
C19	19 carbons
CAM	complementary alternative medicine
CAT	catalase
CCNE1	G1/S-specific cyclin-E1
CDC	center for disease control and prevention
CLL	chemokine (C-C motif) ligand 2
COVID	coronavirus disease 2019
COX-2	cyclooxygenase-2
Cu^2^+	copper (II) cation
CYP	cytochrome P450
DNA	deoxyribonucleic acid
DU245	cell line from human prostate cells
EC	epicatechin
ECG	epicatechin gallate
EGC	epigallocatechin
EGCG	epigallocatechin gallate
ER	estrogen receptor
ERRγ	estrogen-related receptor gamma
ERK	extracellular signal-regulated kinases
FDA	Federal Drug Administration USA
Fe^3^+	ferric ion
FGF	fibroblast growth factor
FOXM1	forkhead box protein M 1
FT3	free triiodothyronine
FT4	free thyroxine
GABA	gamma-aminobutyric acid
GAL	galanin
GF	growth factor
GH	growth hormone
GI	gastrointestinal
GPR30	G protein-coupled receptor 30
GPER	G protein-coupled estrogen receptor
GRAS	generally recognized as safe
HDL	high density lipoprotein (cholesterol)
HDS	herbal dietary supplement
HIFα	hypoxia-inducible factor 1-alpha
HMP	human microbiome project (NIH)
HO1	hemeoxygenase-1
HS	health survey
HSE	hexanic *S. repens* (saw palmetto) extract
IC50	half maximal inhibitory concentration
IGF-1	insulin-like growth factor 1
IGF1R	insulin-like growth factor receptor
IGFBP	insulin-like growth factor binding protein
IIEF	international index of erectile function
IL	interleukin
INF	interferon
IFNγ	interferon-gamma
IR	insulin receptor
IPSS	international prostate symptom score
JAK	janus kinases
JUK	c-jun n-terminal kinase
LNCap	metastatic lymph node lesion of human prostate cancer (cell line)
LUTS	lower urinary tract symptoms
MAPK	mitogen activated protein kinase
MCP1	monocyte chemoattractant protein
MCM	mini-chromosome maintenance
MDA	malodialdehyde
MetabS	metabolic syndrome
MMP	matrix metalloproteinase
MVD	micro-vessel density
NFkβ	nuclear factor Kappa B
NGS	next-generation sequencing
NIH	National Institutes of Health USA
iNOS	inducible nitric oxide synthase
Nrf2	nuclear factor erythroid 2-related factor 2
NO	nitric oxide
P53	tumor protein
PI3K	phosphoinositide 3-kinases
PSA	prostate-specific antigen
PSE	pumpkin seed extract
PVR	post-void residual urinary volume
P38	mitogen-activated protein kinases
PCNA	proliferating cell nuclear antigen
PBX	prostate histology index
QOL	quality of life
Qmax	peak urinary flow rate
RAF	rapidly accelerated fibrosarcoma- kinases
RNA	ribonucleic acid
ROS	reactive oxygen species
RTKN2	rhotekin-2
SERM	selective estrogen receptor modulator
SHBG	sex hormone binding globulin
Smad	family- structurally similar proteins, signal transducers for receptors of TGF-B
SOD	superoxide dismutase
SPE	saw palmetto extract
SPO	conventional saw palmetto
STAT	signal transducer and activator of transcription proteins
TGFβ	transforming growth factor beta
TH	thyroid hormone
TR	thyroid receptor
TRAMP	transgenic adenocarcinoma of the mouse prostate
TRE	thyroid hormone response element
TSH	thyroid stimulating hormone
TNF-α	tumor necrosis factor alpha
TTK	dual specific protein kinase TTK
TURP	transurethral resection of the prostate
US	United States
VCAM-1	vascular cell adhesion molecule 1
VEGF	vascular endothelial growth factor
VISPO	enriched saw palmetto
WS PRO	herbal prostate supplement
